# Defining lower limits of biodegradation: atrazine degradation regulated by mass transfer and maintenance demand in *Arthrobacter aurescens* TC1

**DOI:** 10.1038/s41396-019-0430-z

**Published:** 2019-05-09

**Authors:** Kankana Kundu, Sviatlana Marozava, Benno Ehrl, Juliane Merl-Pham, Christian Griebler, Martin Elsner

**Affiliations:** 1 Institute of Groundwater Ecology, Helmholtz Zentrum München, Ingolstädter Landstraße 1, Neuherberg, Munich, Bavaria D-85764 Germany; 2 Core Facility Proteomics, Helmholtz Zentrum München, Heidemannstr 1, Munich, D-80939 Germany; 3 University of Vienna, Center of Functional Ecology, Division of Limnology, Althanstrasse 14, Vienna, 1090 Austria; 4 Department of Analytical Chemistry and Water Chemistry, Technical University of Munich, Marchioninistrasse 17, Munich, D-81377 Germany

**Keywords:** Environmental microbiology, Environmental sciences

## Abstract

Exploring adaptive strategies by which microorganisms function and survive in low-energy natural environments remains a grand goal of microbiology, and may help address a prime challenge of the 21st century: degradation of man-made chemicals at low concentrations (“micropollutants”). Here we explore physiological adaptation and maintenance energy requirements of a herbicide (atrazine)-degrading microorganism (*Arthrobacter aurescens* TC1) while concomitantly observing mass transfer limitations directly by compound-specific isotope fractionation analysis. Chemostat-based growth triggered the onset of mass transfer limitation at residual concentrations of 30 μg L^−1^ of atrazine with a bacterial population doubling time (*t*_d_) of 14 days, whereas exacerbated energy limitation was induced by retentostat-based near-zero growth (*t*_d_ = 265 days) at 12 ± 3 μg L^−1^ residual concentration. Retentostat cultivation resulted in (i) complete mass transfer limitation evidenced by the disappearance of isotope fractionation (ε^13^C = −0.45‰ ± 0.36‰) and (ii) a twofold decrease in maintenance energy requirement compared with chemostat cultivation. Proteomics revealed that retentostat and chemostat cultivation under mass transfer limitation share low protein turnover and expression of stress-related proteins. Mass transfer limitation effectuated slow-down of metabolism in retentostats and a transition from growth phase to maintenance phase indicating a limit of ≈10 μg L^−1^ for long-term atrazine degradation. Further studies on other ecosystem-relevant microorganisms will substantiate the general applicability of our finding that mass transfer limitation serves as a trigger for physiological adaptation, which subsequently defines a lower limit of biodegradation.

## Introduction

Adaptive strategies of bacteria to cope with extremely low-energy input are of utmost importance to understand life on earth and in extreme environments [1], yet remain an underexplored field of microbiology and environmental biotechnology. Of particular relevance is the degradation of man-made chemical contaminants such as pesticides and pharmaceuticals in the environment [2, 3]. An increasing number of anthropogenic chemicals are detected in natural ecosystems such as ground- and surface water at low concentrations exceeding typical drinking water thresholds (0.1 μg L^−1^). A telling example is atrazine, an s-triazine herbicide: although the use of atrazine was banned in Germany almost 30 years ago, it is still the most frequently detected contaminant in German groundwater [4]. From atrazine-catabolizing microbial populations and monocultures, it is well-established that atrazine can be degraded [5–7], and that degradation may involve two different pathways: either by oxidative dealkylation leading to the intermediate formation of desethyl- and desisopropylatrazine, or by hydrolysis to form the intermediate dehalogenated product hydroxyatrazine (Supplementary Fig. S1) [5, 8]. Finding out why a compound that is in principle biodegradable [9] appears to be persistent at low concentrations (μg L^−1^) remains an unresolved question [10]. Typical regulatory tests, and most laboratory-based degradation tests have been performed at higher concentrations (mg L^−1^) [11] and agricultural studies investigate pulses creating “feast and famine conditions” [12]. In groundwater, in contrast, microorganisms face continuous exposure to concentrations (e.g., atrazine over 0.5 μg L^−1^) that are of similar magnitude as average concentrations of dissolved readily degradable organic carbon, or amino acids (10 and 1 μg L^−1^, respectively) [13, 14]. To develop management strategies fostering natural attenuation of micropollutants, it is, therefore, important to understand the limits of biodegradation at low concentrations, and to explore the underlying drivers behind them.

Two particular drivers are debated to lie at the heart of such a limited degradation. Competing paradigms claim that either (i) mass transfer (uptake into microbial cells) limits otherwise rapid enzymatic transformation; [15] or that (ii) physiological adaptation is responsible meaning that microorganisms switch to maintenance mode and downregulate their catabolic enzymes when—below a certain low concentration—extensive degradation appears to be no longer energetically favorable. Even though complementary studies with microbial communities highlight a link between degradation capacity and community structure [16, 17], in a dedicated study on adaptation, it is reasonable to exclude confounding complexities and to focus on the simplest case: a single species known to degrade the micropollutant in question. *Arthrobacter aurescens* TC1 is a potential candidate because it metabolizes atrazine through a hydrolytic pathway, which is wide-spread in microorganisms [5]. Moreover, it can grow on atrazine both as C and N source and can degrade other s-triazine herbicides detected in the environment [5, 18]. In a previous experiment, we cultivated *A. aurescens* TC1 in chemostats, and compound-specific isotope fractionation analysis indicated that mass transfer through the cell membrane became limiting at substrate concentrations of 30 μg L^−1^ [19]. Seminal work by Egli and others [20–22], have observed residual concentrations at about the same range of 30–100 μg L^−1^ in organic compound degradation in chemostat experiments. However, 30 μg L^−1^ is still 3–10 times higher than typical concentrations found in groundwater populated with 10^5^ bacterial cells mL^−1^ [23]. Hence, microorganisms must be able to both metabolize and gain energy for basic cell functions even at lower carbon concentrations [13]. This calls for exploring the other end member—the bottleneck imposed by the physiology of bacterial adaptation at low concentrations.

Degradation at low concentrations is defined by the maintenance energy (*m*_E_), i.e., the requirement to maintain a cell’s viability: sustaining the proton motive force, osmoregulation, degradation of macromolecules, protein, and RNA repair, etc. [24]. Chemostats offer an opportunity to manipulate the microbial growth rates so that physiology and m_E_ can be studied at a constant growth rate and constant steady-state concentration [25]. However, in natural ecosystems microorganisms remain metabolically active at extremely low (near-zero) growth rates, which cannot be mimicked in a chemostat since the growth is necessary to balance biomass wash-out. Retentostat experiments, in contrast, provide conditions of extremely low-growth rates and low substrate conversion. This is done via retaining of biomass: at constant substrate addition cells proliferate and the amount of substrate per bacterial cell decreases with time. Retentostat cultivation has been recognized to create a “twilight” between growth and stationary phase with interesting properties for secondary metabolite production in biotechnological research [26]. In environmental research, in contrast, retentostat studies are limited, and only a few have been conducted into metabolic activity in organisms relevant to a specific environmental question [27, 28]. Most available studies focused on sugars, which are very different from organic pollutants because they follow fast degradation kinetics and are synthesized endogenously. Hence, these studies cannot mimic the degradation of organic micropollutants in aquatic environments.

Our contribution aims to fill this research gap. Chemostat and retentostat experiments were conducted with *A. aurescens* TC1 grown on atrazine. Our recent finding stresses the particular relevance of the investigated concentration range: that mass transfer limitation appears to define the onset of morphological adaptation [19]. Here, we follow-up on this intriguing evidence and explore both the nature of bacterial adaptation and the degree of mass transfer limitation when further decreasing concentrations in retentostat experiments to mimic an endpoint of exacerbated energy limitation. To detect mass transfer limitation, we measured observable isotope fractionation. To investigate the nature of physiological adaptation, *m*_E_ were experimentally determined both in chemostat and retentostats, and bacterial adaptation was investigated by flow cytometry and comparative proteomics.

## Materials and methods

### Strain, growth condition, and media

The soil bacterium *A. aurescens* TC1 [9], was grown on mineral salt medium (MS) supplemented with 30 mg L^−1^ atrazine (Cfm Oskar Tropitzsch, Germany) as a sole source of C, N, and energy. The medium was prepared in MilliQ^®^ water with a total organic carbon content (<10 μg L^−1^) three orders of magnitude lower than the atrazine feed, and pH was adjusted to 7.2 with sodium hydroxide (1.0 M). The medium was autoclaved at 121 °C for 20 min and cooled. Later, powdered atrazine was added and stirred vigorously for 48 h to facilitate dissolution. Subsequently, FeCl_3_.6H_2_O solution (5.14 mg L^−1^) was added, and the medium was filtered (0.22 μm) to remove any solid atrazine residue. To prepare the pre-culture for chemostat and retentostat cultivation, *A. aurescens* TC1 was grown on MS media with excess atrazine in a shaken flask until an optical density at 600 nm (OD_600_) of 0.1 was reached.

### Chemostat cultivation

Chemostat cultivations of *A. aurescens* TC1 were performed in duplicate 3-L bioreactors (Applikon Biotechnologie B.V., The Netherlands). Bioreactors were equipped with pH, aeration, temperature, level, and agitation controls by myControl^®^ (Applikon Biotechnologie B.V., The Netherlands). An *A. aurescens* TC1 pre-culture (10% (v/v)) was used for inoculation. The agitation speed was 800 rpm and O_2_ was maintained at 50% of saturation throughout cultivation with an air or nitrogen flow of 0.1 L gas L^−1^ min^−1^ as required. Also, the working volume of the bioreactor was maintained at 2000 mL by a level controller, the pH was held constant at 7.2, and the temperature was 25 °C. No foaming was observed eliminating the necessity of adding an antifoaming agent. The bioreactors were operated at eight dilution rates (D) [defined as the ratio of the medium flow rate (mL h^−1^) and culture volume (L)] of 0.068, 0.056, 0.048, 0.032, 0.023, 0.018, 0.009, and 0.006 h^−1^. Dilution rates were changed after achieving a steady-state at a particular D meaning that culture parameters such as cell concentration, atrazine, and 2-hydroxyatrazine remained constant (<5 and <10% relative variation, respectively) for at least five reactor volume changes.

### Retentostat cultivation

Duplicate retentostat experiments were performed at a dilution rate of 0.02 h^−1^ in identical bioreactors as in the chemostat experiments and with identical operating conditions, with the exception that reactors were in addition equipped with an autoclavable polyethersulfone cross-flow filter with a pore size of 0.22 μm (Flownamics, USA) to retain biomass in the reactor. The filtration unit was installed via a head plate port to the bioreactors. An internal sterile filtration loop was established with a connection of the level sensor to the myControl® and a peristaltic pump to allow filtration of effluent during level control throughout the cultivation.

### Measurement of biomass, substrate, and metabolites

During chemostat and retentostat cultivations, samples were withdrawn from the bioreactors by a sterile sampling loop (Applikon Biotechnologie B.V., The Netherlands). The sampling volume was kept below 3% of the working volume during routine sampling and below 10% under steady-state conditions to minimize the disturbance caused by withdrawal. After filtering the samples, concentration measurements of atrazine and 2-hydroxyatrazine by high -performance liquid chromatography (HPLC) were performed as detailed in the Supporting Information. To measure the cell dry weight, samples were centrifuged at 4 °C in a pre-weighed tube washed with 0.9% NaCl and dried at 85 °C to constant weight [29].

### Estimation of cell numbers, viability, and morphology

Cells were fixed with 2.5% glutaraldehyde and stored at 4 °C for a maximum of 20 days before measurement. The cells were stained with SYBR Green I and propidium iodide to estimate the number of total cells and the fraction of viable cells respectively. For morphology, the fixed cells were analyzed on agar glass slides by light microscopy with an Axioscope 2 Plus microscope (Carl Zeiss AG, Germany) [30]. Details of the analysis are provided in the Supporting Information.

### Compound-specific isotope analysis of atrazine in retentostat

At the end of the retentostat cultivation, samples (1000 mL) were withdrawn from the two bioreactors through 0.22 μm filters. Atrazine was extracted with dichloromethane. Carbon isotope analysis of atrazine was performed on a GC-IRMS system consisting of a TRACE GC Ultra gas chromatograph (Thermo Fisher Scientific, Italy) equipped with a DB-5 analytical column (60 m, 0.25 mm ID, 1.0 μm film, Agilent Technologies, Germany) coupled to a Finnigan MAT 253 isotope ratio mass spectrometer via a Finnigan GC Combustion III interface (both Thermo Fisher Scientific, Germany). The isotope fractionation (ε) was determined as the difference of the isotope values of inflow and outflow. Details of the analysis are described in the Supporting Information.

### Estimation of growth kinetics parameters

#### Chemostat

The mass balance for cell concentration (*C*_x_) and residual substrate concentration (*C*_s_) in the chemostat cultivation were calculated as:

1 $$\frac{{dC_x}}{{dt}} = \mu \cdot C_x - D\cdot C_x$$

2 $$\frac{{dC_s}}{{dt}} = D\cdot \left( {C_{s,in} - C_s} \right) - q_s\cdot C_x$$

where *q*_s_ is the biomass-specific substrate (atrazine) consumption rate, *C*_*s,in*_ is the substrate concentration in the media and *μ* is the specific growth rate. The consumption rate *q*_s_ was described by a Michaelis–Menten type expression.

3 $$q_s = \frac{{q_s^{max}\cdot C_s}}{{K_s + C_s}}$$

where *K*_s_ and $q_s^{max}$ are Monod affinity constant and maximal substrate consumption rate. The experimentally measured *q*_s_ at each dilution rate at steady-state was used to fit the model (Eq. 3) to estimate *K*_s_ and $q_s^{max}$. To estimate the maintenance demand *m*_s_, a modified Herbert–Pirt equation was used [31, 32].

4 $$\mu = Y_{x/s}^{max}\cdot \left( {q_s^{max} \cdot \frac{{C_s}}{{C_s + K_s}} - m_s} \right)$$

where $Y_{x/s}^{max}$ denotes the (hypothetical) maximum growth yield in case all consumed substrate is channeled to biomass.

#### Retentostat

The dynamic biomass concentration C_x_(t) during retentostat cultivation was described by the Van Verseveld equation [33]:

5 $${\mathrm{C}}_x\left( {\mathrm{t}} \right) = {\mathrm{C}}_{x,0}\cdot e^{ - m_s\cdot Y_{\frac{x}{s}}^{max}\cdot t} + \frac{{D\cdot \left( {C_{s,in} - C_s} \right)}}{{m_s}}\left( {1 - e^{ - m_s\cdot Y_{\frac{x}{s}}^{max}\cdot t}} \right)$$

The biomass growth rate was calculated as:

6 $$\mu = \frac{{dC_x^{Total}{\mathrm{/}}dt}}{{C_x^{viable}}}$$

The model implementation, fitting parameter estimations and model analysis was performed using Python and employing the built-in functions in scientific libraries NumPy and SciPy [34]. The calculated *m*_s_ was converted to maintenance energy (*m*_E_) based on Δ_*R*_G^0′^_cat_ (Gibbs free energy released during substrate catabolism) as described by Tijhuis et al. [25]. Details are provided in the Supporting Information.

#### Proteomic analysis

Samples were withdrawn from the chemostats at steady-state and the end of the retentostat cultivation. Since cell densities were about 10 times higher in retentostats, cell pellets were made from 200 mL sampling volume in chemostats and 20 mL in retentostats for protein extraction (for details see Supporting Information). In total, 10 μg of whole protein extract from each sample were used for trypsin digestion using a modified FASP procedure [35]. Liquid chromatography coupled with mass spectrometry (LC-MS/MS) analysis was performed on a QExactive HF mass spectrometer (Thermo Fisher Scientific) online coupled to an Ultimate 3000 RSLC (Dionex). The acquired spectra were loaded to the Progenesis QI software (version 3.0, Nonlinear Dynamics, part of Waters) for label-free quantification and analyzed as described previously [36]. All MS/MS spectra were exported as Mascot generic file and used for peptide identification with Mascot (version 2.5.1) in the UniProt *A. aurescens* TC1 protein database (1,514,995 residues, 4566 sequences). Search parameters used were: 10 ppm peptide mass tolerance and 0.02 Da fragment mass tolerance, one missed cleavage allowed, carbamidomethylation was set as fixed modification, methionine oxidation and asparagine or glutamine deamidation were allowed as variable modifications. A Mascot-integrated decoy database search calculated an average false discovery of 0.48% when searches were performed with the mascot percolator algorithm and *p* < 0.05. Peptide assignments were re-imported into the Progenesis QI software, and the abundances of all peptides allocated to each protein were normalized and summed up. This normalization allowed all samples to be on the “same scale” by applying a global scaling factor between the samples. One of the sample runs was automatically selected as normalization reference [37]. Progenesis normalization method is based on the peptide ion ratio calculation in between the sample and normalized reference. This removes the influence of absolute abundance from the process, which is an advantage over total-abundance-based methods.

### Statistical analysis

Normalized and log_2_-transformed protein abundances were used to identify differentially abundant proteins using a linear model for microarray data analysis (LIMMA) in Bioconductor [38, 39]. The undetected proteins or the missing values were handled in the same way as for linear models, i.e., rows with missing values are subjected to na.exclude, or “case-wise deletion” [38]. During pair-wise comparison between different conditions in chemostats and retentostats, only proteins showing a log fold change higher than log_2_(2.5) and a Benjamini–Hochberg [40] corrected *p*-value of <0.05 were considered to be differentially abundant. Normalized protein abundance of each protein was converted to *z-*score by using the transformation [*x*-mean(*x*_a…n_)]/SD(*x*_a…n_), where *x* is one protein in the data set population (a…n) and SD is the standard deviation. Hence, *z*-score calculates how many SD units a protein’s abundance is away from the mean abundance derived from all conditions (population mean). The probability distribution of the *z*-scores of all proteins in different conditions was illustrated as violin plots created using the seaborn package [41]. Hierarchical clustering of the *z*-scores for the differentially abundant proteins was performed using “Euclidean distance” as a distance function and was visualized as heatmaps with the seaborn package.

## Results

### Chemostat cultivation of *A. aurescens* TC1 indicates a high atrazine degradation capability and a high maintenance demand under growth conditions

**Fig. 1:**
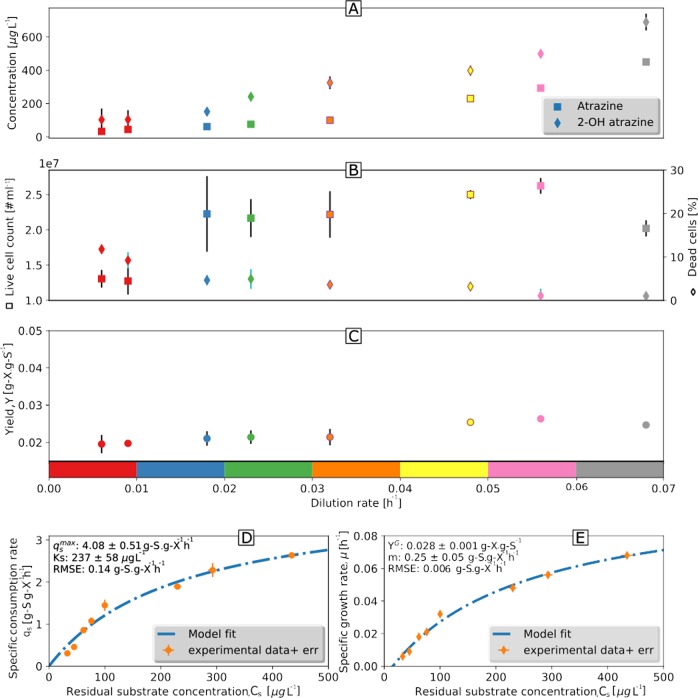
Growth parameters of *A. aurescens* TC1 plotted versus dilution rates indicate a high atrazine degradation capability under oligotrophic conditions and high maintenance demand. **a** Concentration of residual atrazine and the first metabolite, 2-hydroxyatrazine. **b** Live cell numbers per milliliter and the percentage of dead cells. **c** Yield. **d** The symbols indicate the relationship between the biomass-specific atrazine consumption rate (*q*_s_) and residual substrate concentration at different dilution rates. The line indicates *q*_s_ according to a Michaelis–Menten type relationship (Eq. 3) using fitted growth parameters of maximum specific atrazine consumption rate ($q_s^{max}$) and Monod affinity constant (K_s_). **e** The specific growth rates (μ) dependence on substrate concentrations obtained experimentally is shown by symbols and the line indicates the same relationship according to a modified Herbert–Pirt model (Eq. 4) using fitted maintenance demand (*m*_s_) and maximum growth yield *Y*^*G*^. Data points represent the mean ± standard deviation of replicates. RMSE: root mean square error

Chemostat cultivation of *A*. *aurescens* TC1 resulted in >99% degradation of *C*_*atrazine,in*_ = 30 mg L^−1^ to cyanuric acid at all investigated dilution/growth rates: D_high_ (0.068, 0.056, and 0.048 h^−1^), D_medium_ (0.032 and 0.023 h^−1^), and D_low_ (0.018, 0.009, and 0.006 h^−1^) (Fig. 1a). Lower D increased substrate and biomass residence time resulting in decreasing concentrations of atrazine and its direct metabolite 2-hydroxy-atrazine (2-OH atrazine) according to a classic chemostat behavior (Fig. 1a). Cell concentrations did not change significantly from D_high_ to D_medium_ (2.2–2.69 × 10^7^ cells mL^−1^, Fig. 1b), but dropped to 1.31 × 10^7^ cells mL^−1^ at D_low_ (0.006 and 0.009 h^−1^, Fig. 1b). Remarkably, even at D_low_ the viability, as determined by specific staining and flow cytometry, remained as high as 90% (Fig. 1b). No significant difference in biomass yield (*Y*_x/s_) was observed (Fig. 1c). At higher D, *q*_s_ (Eq. 2) increased (Fig. 1d, Supplementary Table S1) reflecting a high atrazine degradation capability of *A. aurescens* TC1. Equation 3 was fitted using measured *q*_s_ and *C*_s_ to estimate $q_s^{max}$ and *K*_s_ as 4.08 ± 0.51 g S (g X)^−1^ h^−1^ and 237 ± 58 μg L^−1^ (~1 μM), respectively. The collinearity index (Eq. 8, Supporting Information) was sufficiently low (4.80) to exclude significant interrelation between the parameters $q_s^{max}$ and *K*_S_.*m*_s_ was calculated by fitting atrazine concentrations at different D according to Eq. 4 (Fig. 1e). The resultant *m*_s_ was 0.25 g S (g X)^−1^ h^−1^, which translates into *m*_E_ = 97 ± 10 kJ (C-mol biomass)^−1^ h^−1^ based on Δ_*R*_G^0′^_cat_ = −3338 kJ mol^−1^ for mineralization of the amine side chains in atrazine (see Supporting Information).

### Retentostat cultivation results in lower maintenance demand implying adaptation of *A. aurescens* TC1 at a near-zero growth rate

**Fig. 2:**
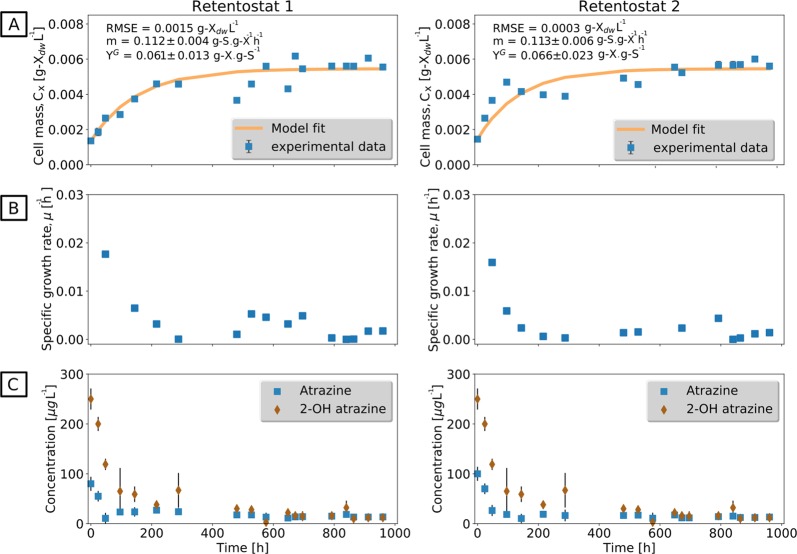
Retentostat cultivation reveals a different maintenance energy requirement of *A. aurescens* TC1 at near-zero growth rate in continuous culture with 100 % biomass retention. **a** Measured biomass concentration (gram dry weight (gdw) L^−1^) in retentostat 1 and 2 over time. Data points represent the mean ± standard deviation of duplicate samples. The line indicates the biomass calculated with the fitted van Verseveld equation (Eq. 5). The fitted maintenance requirement (m) is two times lower than in chemostat. **b** Calculated specific growth rates, μ (h^−1^) over time. At the end of the experiment, near-zero growth condition was achieved corresponding to 225 days doubling time. **c** Concentration of residual atrazine and its metabolite 2-OH atrazine were lower than in the chemostat experiment. Bars indicate the standard deviation of replicates. RMSE : root mean square error, *Y*^*G*^ : maximum growth yield

**Fig. 3:**
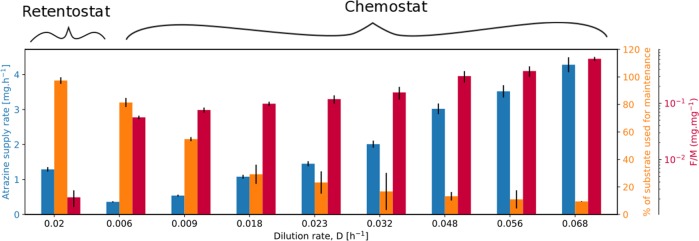
Allocation of energy fluxes to maintenance and food to microorganisms (F/M) ratio during chemostat and retentostat cultivation

To explore the adaptation of *m*_E_ to extremely low-growth rates that would rather mimic conditions in natural ecosystems, retentostat cultivations were performed. Two independent retentostat cultivations were successfully conducted for 42 days (Fig. 2) at a D of 0.020 h^−1^. Wall growth or filter clogging—typical problems of retentostat experiments over longer time periods—were not observed. During the first 18 days of cultivation, the biomass (grams dry weight (g_dw_) L^−1^) increased linearly (growth phase) until a cell concentration of 2.79 × 10^8^ cells mL^−1^ was reached at which the atrazine supply rate met the *m*_E_ requirement of the cells (Fig. 2a). In this period, μ decreased from 0.018 to 0.005 h^−1^ and, finally, reached a level of 0.0001 h^−1^ after 42 days corresponding to a doubling time of *t*_d_ = 225 days (Fig. 2b). During this time, 97% of the available energy flux was directed to the maintenance of basic cell functions and the energy availability for biomass as indicated by a ratio of food/microorganism (F/M) that was much lower than in chemostat (Fig. 3, Supplementary Table S1). After the growth phase (22 days), very high viability was observed (99.5 ± 0.5%) implying that the lysis of dead cells can be ruled out as a significant source of energy for bacterial metabolism. Accordingly, no cell debris was observed during microscopic observation of the retentostat culture. Biomass accumulation in the retentostat cultivations was modeled with the van Verseveld equation (Eq. 5) to estimate *m*_*s*_ and $Y_{x/s}^{max}$ (collinearity index = 1.30). The estimated *m*_s_ was 0.11 g S (g X)^−1^ h^−1^ corresponding to *m*_E_ = 37 ± 8 kJ (Cmol biomass)^−1^ h^−1^, which is ~2 times lower than in chemostats. This implies that *A. aurescens* TC1 undergoes further adaptation to lower its *m*_E_ requirement under exacerbated low-energy conditions. The concentration of atrazine and 2-OH atrazine in retentostats was 12 ± 3 and 10 ± 5 μg L^−1^, respectively, at the end of the cultivation (Fig. 2c). This extracellular atrazine concentration, therefore, represents an experimentally determined minimum substrate concentration (*S*_min_) observed at quasi-zero growth conditions where the substrate flux is just about sufficient to meet the cell’s *m*_E_ demand for long-term viability.

### Isotope fractionation reveals full mass transfer limitations in retentostats

Compared with isotope fractionation in chemostats at D_high_ (ε^13^C = −5.36‰ ± 0.20‰ at µ = 0.023 h^−1^) isotope fractionation in retentostats essentially disappeared (ε^13^C = −0.45‰ ± 0.36‰) falling behind the value of −4.34‰ ± 0.13‰ (±SEM, *N* = 2) that indicated the onset of mass transfer limitations in chemostats at D_low_ 0.018 h^−1^ in our recent study [19]. This provides unequivocal evidence that mass transfer, not the enzyme reaction, was rate limiting. Otherwise, the isotope effect of the enzyme reaction (ε^13^C = −5.4‰) would be fully observable [42, 43]. The retentostat cultivation, hence, represented a system where full mass transfer limitation could be experimentally verified. The same line of evidence—isotope fractionation—pinpointed chemostats at D_high_ as systems where mass transfer was not rate limiting. Since bacteria persist long enough in both systems to adapt to the prevailing conditions, our experiments offer a unique opportunity to study bacterial adaptation between conditions of no, partial, and full mass transfer limitation.

### Physiological adaptation in morphology

**Fig. 4:**
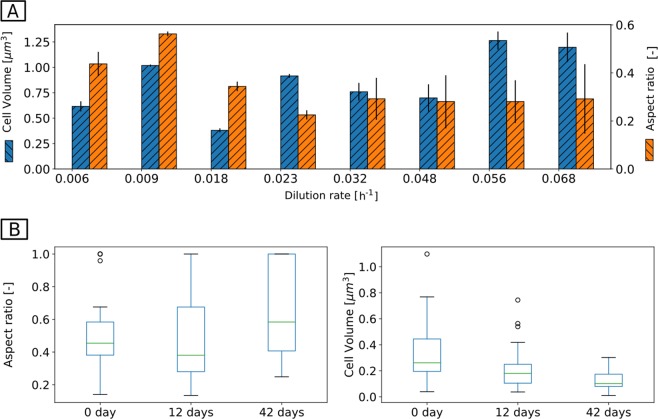
Morphological adaptation to limiting substrate conditions in the chemostats (**a**) and in retentostat (**b**). In the chemostats, at low-growth rates cells were smaller rods in comparison with high growth rates. In the retentostat, a transition from rod (0 d) to coccus shape was observed after 42 days at near-zero growth condition. This morphological change increases the surface-to-volume ratio of the cells maximizing the interface for transport of substrates and nutrients. Data points show the average from the evaluation of two biological replicates of chemostats and one replicate in the case of the retentostat (R1 in Fig. 2)

In chemostats, cells were rod shaped at all D. However, shifts in morphology were observed when D was changed (Fig. 4a). At D_high_, cells were long rods with a smaller aspect ratio (width/length) compared with cells at D_low_. This equates to a drop in cell volume from 1.28 μm^3^ at D_high_ to 0.68 μm^3^ at D_low_.

In retentostats, cells were mainly rod shaped at the beginning of cultivation, whereas both coccoid and shorter rod-shaped cells emerged toward the end of cultivation (Fig. 4b, Supplementary Fig. S2). This transition in morphology was first observed after 12 days as reflected by a smaller aspect ratio and a smaller volume of the cells. The morphologically changed cells dominated (*>*50%) at the end of the cultivation (42 days).

### Physiological adaptation at the proteome level

**Fig. 5:**
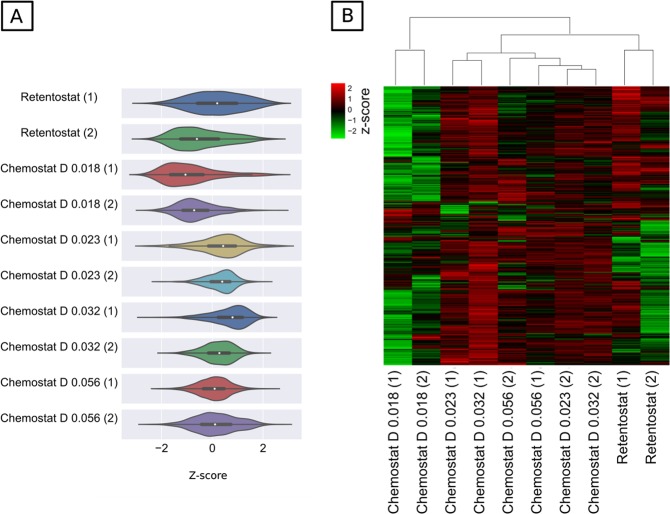
Physiological changes associated with different energy settings under mass transfer limitation in chemostats and retentostats. **a** The probability distribution of *z*-score-normalized abundances of 1594 proteins at different dilution rates in chemostat and retentostat shown as violin plots. Violin shape: kernel density curves; white node in center: median; black box inside the violin: box-and-whisker plot. **b** Heat map representing the clustering of 671 significantly abundant proteins at dilution rates 0.018, 0.023, 0.032, 0.056 h^−1^ and in retentostat cultivation. Protein abundance is displayed in the heat map as *z*-scores (i.e., calculated based on how many SD units a protein’s abundance is away from the mean abundance derived from all conditions) in the range between 2 (of significantly higher abundance, red) and −2 (of significantly lower abundance, green). D: dilution rates, SD: standard deviation. Each chemostat and retentostat cultivation was performed in replicates as indicated by dilution rates in the brackets below the heat map

The proteome state of the cells was analyzed at D_low_ (0.018 h^−1^), D_medium_ (0.023 h^−1^), and D_high_ (0.032 and 0.056 h^−1^) in chemostats and retentostats at the end of the cultivation. In total, 1733 proteins were quantified (~37% coverage of total predicted protein-coding genes in *A. aurescens* TC1),  of which 1594 proteins were quantified in all the samples (Supplementary Tables S2, S3). The distribution of *z*-score normalized overall relative protein abundances is illustrated in a violin plot (Fig. 5a). The median *z*-score at D_medium_ (0.023 h^−1^) and D_high_ (0.032, and 0.056 h^−1^) under no mass transfer limitation was in between 0.5 and 1.2. At D_low_ 0.018 h^−1^ (partial mass transfer limitation), the median of *z*-score distribution was in between −1.3 and −1.6 indicating a high number of proteins with low abundances. At near-zero growth rate in retentostats (full mass transfer limitation), the median *z*-score varied between 0.02 and −0.2 with a broad distribution suggesting that in contrast to D_low_ retentostats also had some proteins with high abundances. Non-parametric clustering for all proteins across all cultivation conditions shows three distinct clusters for (D_high_ + D_medium_), D_low_, and retentostats (Supplementary Fig. S3). Hence, (D_high _+ D_medium_) was treated as one group and compared with proteins detected in the other two groups—D_low_ and retentostats. Hierarchical clustering of differentially abundant proteins (671) in at least one pair-wise comparison, shows that many proteins that had a significantly higher abundance (*z*-score around 2) at (D_high_ + D_medium_) were less abundant at D_low_ and retentostats suggesting a clear difference in physiology (Fig. 5b). To interpret implications for metabolism, all proteins were linked to clusters of orthologous groups categories (COG) (Supplementary Fig. S4) [44].

### Atrazine catabolic pathway

**Fig. 6:**
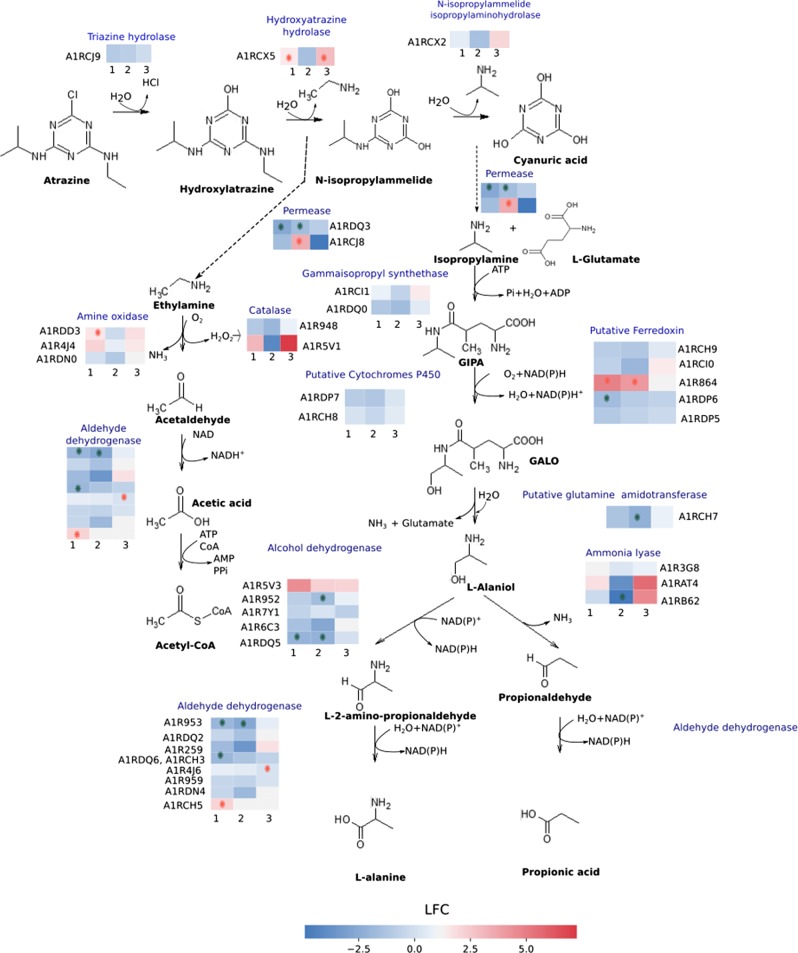
Atrazine degradation pathway adapted from the literature [5, 46, 68]. The relative abundance of proteins (see Table 1) is indicated in the colored rows, where multiple putative proteins catalyzing the same step are indicated as multiples rows. Three pair-wise comparison groups were created as indicated by the numbers 1, 2, 3. 1: RET vs. CHEM; 2: D0.018 vs. CHEM; 3: RET vs. D0.018. RET represents retentostats under full mass transfer limitation. CHEM represents the combined data from chemostats at D_high_ + D_medium_—0.023, 0.032, 0.056 h^−1^ (under no mass transfer limitation). D0.018 represents chemostats at D_low_—0.018 h^−1^ where isotope fractionation indicated the onset of mass transfer limitation. Different colors represent the log fold change (LFC) in specific proteins from a pair-wise comparison between chemostats at different dilution rates and retentostats. For example, a red field in comparison group 1 means that a protein was in higher abundance in retentostats than in chemostats at 0.023, 0.032, 0.056 h^−1^. Symbols (*) in red indicate proteins of significantly higher abundance and in green of significantly lower abundance in the respective pair-wise comparison, where the criteria for significant differences were a *p*-value of <0.05 together with a cut off log fold change (LFC) of log_2_(2.5). D : dilution rates, GIPA: gamma-glutamyl-isopropylamide, GALO: gamma-glutamyl-l-alaninol

Since substrate limitation may have most immediate consequences for associated catabolic enzymes, proteins related to the atrazine degradation pathway were compared between (D_high_ + D_medium_), D_low_ and retentostat (Fig. 6, Table 1). *A. aurescens* TC1 degrades atrazine by hydrolytic dechlorination, catalyzed by the enzyme triazine hydrolase, TrzN followed by two hydrolytic deamination reactions catalyzed by hydroxyatrazine hydrolase, AtzB, and *N*-isopropylammelide isopropylaminohydrolase, AtzC. These enzymes convert atrazine sequentially to cyanuric acid, which is not further degraded to allophanic acids unlike observed in some other organisms [5, 45]. Nonetheless, the enzymatic triad of TrzN-AtzB-AtzC liberates amines—isopropylamine and ethylamine, which are metabolized and assimilated (Fig. 6, Table 1) [5, 9]. The relative abundance of proteins in the upper pathway to cyanuric acid was not significantly reduced at D_low_ and retentostats relative to (D_high_ + D_medium_). However, AtzB had significantly higher abundances (log fold change (LFC), i.e., log_2_ of absolute fold change > 1.45) in retentostats compared with D_low_ and (D_high_ + D_medium_).

**Table 1:** Proteins predicted to be related to atrazine degradation pathway

UniProt ID	Gene name	Proteins	RET/CHEM		D0.018/CHEM		RET/D0.018	
			LFC	Adj. *p*-value	LFC	Adj. *p*-value	LFC	Adj. *p*-value
A1RCJ9	AAur_pTC10087	TrzN, triazine hydrolase	−0.94	0.087	−0.36	0.422	−0.57	0.404
A1RCX5	AAur_pTC10218	AtzB, hydroxyatrazine hydrolase	1.48	0.025	−0.89	0.089	2.37	0.019
A1RCX2	Aur_pTC10212	AtzC, *N*-isopropylammelide isopropylaminohydrolase	0.61	0.294	−0.81	0.126	1.42	0.084
^2^A1RDQ3	AAur_pTC20216	Amino-acid permease	−2.71	0.004	−1.87	0.020	−0.84	0.311
^1^A1RCG8	AAur_pTC10056	Putative amino-acid permease	−1.34	0.357	3.19	0.0390	−4.53	0.050
^1^A1RCI1	AAur_pTC10069	IpuC, gamma-glutamylisopropylamide synthetase	0.77	0.133	−0.29	0.502	1.06	0.123
^2^A1RDQ0	AAur_pTC20213	IpuC, gamma-glutamylisopropylamide synthetase	−1.10	0.060	−0.87	0.090	−0.23	0.778
^2^A1RDP7	AAur_pTC20210	Putative cytochrome P450	−0.99	0.096	−0.90	0.096	−0.09	0.920
^1^A1RCH8	AAur_pTC10066	Putative cytochrome P450	0.13	0.752	−0.91	0.037	1.04	0.072
^1^A1RCH9	AAur_pTC10067	Ferredoxin	−0.93	0.211	−0.99	0.140	0.05	0.962
^1^A1RCI0	AAur_pTC10068	Ferredoxin reductase	−0.98	0.290	−1.56	0.0838	0.58	0.659
A1R864	AAur_2711	Putative ferredoxin reductase	4.97	0.002	3.13	0.0172	1.84	0.154
^2^A1RDP6	AAur_pTC20209	Ferredoxin	−1.85	0.038	−0.96	0.170	−0.89	0.387
^2^A1RDP5	AAur_pTC20208	Putative ferredoxin reductase	−1.22	0.025	−0.76	0.086	−0.46	0.454
^1^A1RCH7	AAur_pTC10065	Putative glutamine amidotransferase	−1.33	0.060	−1.48	0.038	0.14	0.894
A1R5V3	AAur_1866	Putative alcohol dehydrogenase	2.79	0.072	2.84	0.056	−0.05	0.980
A1R952	AAur_3063	Alcohol dehydrogenase	−0.79	0.132	−1.61	0.018	0.82	0.223
A1R7Y1	AAur_2625	Alcohol dehydrogenase	−0.55	0.222	−0.08	0.849	−0.47	0.433
A1R6C3	AAur_2040	Alcohol dehydrogenase	−1.76	0.085	−1.47	0.104	−0.29	0.846
^2^A1RDQ5	AAur_pTC20218	Putative alcohol dehydrogenase	−2.07	0.007	−1.25	0.038	−0.82	0.264
A1R953	AAur_3064	Aldehyde dehydrogenase (NAD)	−1.93	0.006	−2.76	0.005	0.83	0.212
^2^A1RDQ2	AAur_pTC20215	Aldehyde dehydrogenase	−0.53	0.378	−0.86	0.118	0.33	0.694
A1R259	AAur_0513	Betaine-aldehyde dehydrogenase	−1.80	0.060	−1.48	0.084	0.32	0.811
^1^A1RCH3; ^2^A1RDQ6	AAur_pTC10061; AAur_pTC20219	Aldehyde dehydrogenase (NAD)	−1.89	0.006	−0.92	0.062	−0.97	0.153
A1R4J6	AAur_1380	Aldehyde dehydrogenase (NAD)	0.71	0.132	−0.75	0.088	1.46	0.047
A1R959	AAur_3070	Aldehyde dehydrogenase	−0.41	0.435	−0.48	0.293	0.07	0.940
^2^A1RDN4	AAur_pTC20196	Aldehyde dehydrogenase	−0.42	0.668	−0.26	0.768	−0.17	0.916
^1^A1RCH5	AAur_pTC10063	Aldehyde dehydrogenase (NAD)	2.16	0.036	−0.50	0.521	2.66	0.057
A1R3G8	AAur_0990	l-serine ammonia-lyase	1.07	0.157	0.70	0.270	0.37	0.727
A1RAT4	AAur_3659	Histidine ammonia-lyase	1.25	0.337	−1.46	0.206	2.72	0.131
A1RB62	AAur_3794	Aspartate ammonia-lyase	−0.17	0.855	−2.59	0.021	2.42	0.065
A1R948	AAur_3059	Catalase	−1.08	0.060	−1.26	0.033	0.18	0.823
A1R5V1	AAur_1864	Catalase	3.37	0.105	−1.36	0.417	4.74	0.081
A1RDD3	AAur_pTC20082	Amine oxidase	1.55	0.006	0.34	0.340	1.21	0.055
A1R4J4	AAur_1378	Amine oxidase	1.97	0.064	0.46	0.590	1.51	0.253
A1RDN0	AAur_pTC20192	Amine oxidase	0.10	0.922	0.18	0.829	−0.08	0.958

Proteins are selected based on atrazine-degrading *Pseudomonas* strain and *Arthrobacter*-related publications [5, 46, 68]. The relative abundances of the proteins are compared between different dilution rates in chemostats and retentostats. Three pair-wise comparison groups were created. 1: RET vs. CHEM; 2: D0.018 vs. CHEM; 3: RET vs. D0.018. RET represents retentostats under full mass transfer limitation. CHEM represents the combined data from chemostats at D_high_ + D_medium_— 0.023, 0.032, 0.056 h^−1^ (under no mass transfer limitation). D0.018 represents chemostats at D_low_— 0.018 h^−1^ (onset of mass transfer limitation). ^1^, ^2^ indicates that the genes encoding for the isopropylamine degradation proteins are present on a plasmid— pTC1 and pTC2, respectively

*LFC:* log fold change, *Adj. p-value:* Benjamini–Hochberg corrected *p*-value

Isopropylamine is transformed into propionic acid and l-alaniol by the Ipu pathway [5, 46]. Remarkably, *A. aurescens* TC1 contains two Ipu pathway gene clusters encoding multiple proteins catalyzing the same step of the pathway (Table 1). First, isopropylamine is transported into the cytoplasm by putative permeases. In the next step, ATP-dependent conversion of isopropylamine and l-glutamate to gamma-glutamyl-isopropylamide is catalyzed by gamma-glutamyl-isopropylamide synthetases, IpuC. A multicomponent cytochrome P450 monooxygenase system catalyzes the conversion of gamma-glutamyl-isopropylamide to gamma-glutamyl-l-alaninol. The next step that liberates l-alaniol and glutamate is postulated to be catalyzed by an amidotransferase enzyme with the same hydrolysis mechanism as IpuF in *Pseudomonas* [46, 47]. Glutamine amidotransferase (AIRCH7) was observed to have significantly lower abundances (LFC = −1.48) in D_low_ relative to (D_high_ + D_medium_).

l-Alaninol is further oxidized by choice of five alcohol dehydrogenases to l-2-amino-propionaldehyde, or deaminated by three possible ammonia lyases to propionaldehyde. Subsequently, l-2-amino-propionaldehyde and propionaldehyde are converted to l-alanine and propionic acid, compounds, which can be processed by reactions of the intermediary metabolism. Most of the proteins in the Ipu pathway were not significantly altered in abundance except for some aldehyde dehydrogenases and glutamine amidotransferases. Interestingly, two of the eight aldehyde dehydrogenases (AIRCH3, A1RDQ5) were significantly less abundant in retentostats while one was highly abundant (AIRCH5, LFC = 2.15). This suggests that alternative isozymes are used at low concentrations. Degradation of ethylamine is initiated by amine oxidases to liberate acetaldehyde, ammonia, and hydrogen peroxide. Out of three amine oxidases postulated to catalyze alkylamine degradation in *A. aurescens* TC1, one protein (A1RDD3) was significantly abundant in retentostats relative to (D_high_ + D_medium_). Acetaldehyde is converted into acetate, by aldehyde dehydrogenase, and further oxidized via the tricarboxylic acid (TCA) cycle or funneled to glyoxylate cycle for carbon assimilation.

### Overview of differentially abundant proteins under mass transfer limitation

**Fig. 7:**
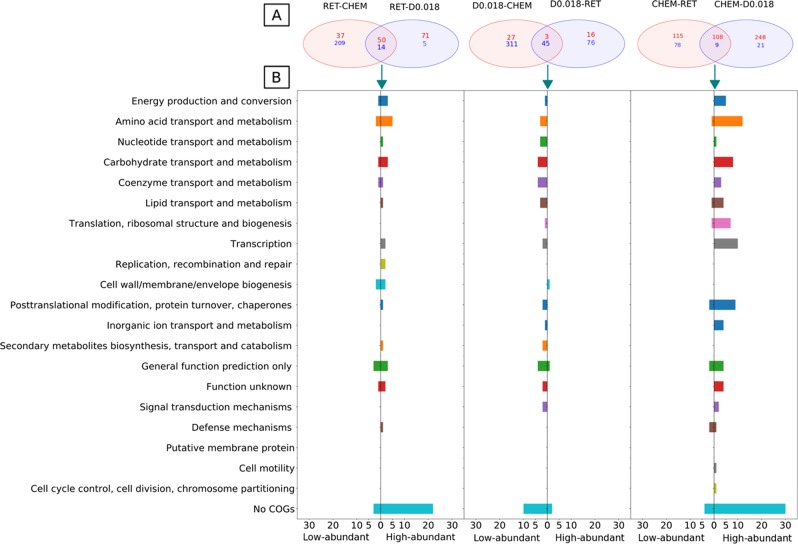
Differentially abundant proteins from pair-wise comparisons between chemostats at different dilution rates and retentostats. Three comparison groups were created: CHEM (chemostats at D_high_ + D_medium_— 0.023, 0.032, 0.056 h^−1^, no mass transfer limitation); D0.018 (chemostats at D_low_—0.018 h^−1^, onset of mass transfer limitation), and RET (retentostat cultivation, full mass transfer limitation). **a** A Venn diagram illustrates differentially abundant proteins between the three groups. The proteins that lie in the intersection of a two-way Venn diagram depicts the overlap of differentially abundant proteins between comparison pairs representing the selection for consideration in more detail in panel **b**. Numbers in red represent significantly high-abundant proteins and those in blue represent significantly low-abundant proteins. **b** Distribution of overlapping significantly high and low-abundant proteins across clusters of orthologous groups (COG) categories in retentostats, D of 0.018 h^−1^ and chemostats (0.023, 0.032, and 0.056 h^−1^)

The three groups corresponding to the respective conditions — chemostats at (D_high + _D_medium_), chemostats at D_low_ and retentostats — were compared with each other. The proteins that lie in the intersection of a two-way Venn diagram depicted the overlap of differentially abundant proteins (*p* < 0.05, cut off LFC = log_2_(2.5)) between comparison pairs (Fig. 7a, Supplementary Tables S4, S5, S6). These differentially abundant shared proteins are hence characteristic of that condition. The distribution of these characteristic proteins of chemostats at (D_high_ + D_medium_), i.e., significantly abundant relative to both D_low_ and retentostats across COG categories (Fig. 7b), shows that most of the highly abundant proteins belong to categories such as amino-acid transport and metabolism, carbohydrate transport and metabolism, energy production, and conversion. Hence, although most of the proteins related to atrazine degradation were not highly abundant in chemostats at (D_high_ + D_medium_) relative to D_low_ and retentostats, abundances of the proteins related to other catabolic pathways were higher. Examples are proteins involved in the conversion of mannose into fructose and in the mannose pathway via phosphomannomutase (A1R321). Besides, cytochrome c oxidase (A1R6U3) involved in the electron transport chain and two components from the pyruvate dehydrogenase complex of the TCA cycle (dihydrolipoamide acetyltransferase A1R5K3 and pyruvate dehydrogenase E1 component A1R7E9) were significantly abundant at (D_high_ + D_medium_). This observation of overall higher abundance of proteins related to catabolism at (D_high_ + D_medium_) is in apparent agreement with a general acceleration of metabolism and cellular processes in response to a high F/M. Proteins for anabolic processes mainly related to the transcription and translational apparatus (e.g., transcriptional regulator of GntR-family (A1R2G6, A1R316), RNA polymerase sigma factor (A1R7Z7), and synthesis of ribosomal proteins (A1R8N1, A1R568)) fell into highly abundant categories in chemostats at (D_high_ + D_medium_). This reflects the reduced building block requirement in the adaptation of the cells to a low F/M at D_low_ (0.018 h^−1^), and retentostats as the synthesis of cellular macromolecules are energy demanding. Although the characteristic proteins of chemostats at (D_high_ + D_medium_) were mostly in the high-abundant category, several less-abundant proteins also stood out. Specifically, glycine betaine transporter (A1R218), streptomycin 6 kinase (A1R510), chaperone protein (A1R5W7), CBS domain proteins (A1R3T7), and cupin domain protein (A1R5W7) were significantly less abundant (LFC > −2.5) relative to both D_low_ and retentostats, which might indicate their role in survival under stress at low concentrations.

The characteristic proteins of D_low_ were mostly in the less-abundant category while in retentostats some of the proteins related to carbohydrate transport and metabolism (phosphoglycerate mutase (A1R3D0), ABC transporters (e.g., A1R438)) also fell into the highly abundant (LFC > 2.1) category. Similarly, some of the amino-acid synthesis proteins such as glutamate synthase (A1R5T1, LFC = 1.7) was also highly abundant in retentostats. Also, proteins playing a role in secondary metabolite biosynthesis — the characteristic feature of near-zero growth condition — were among highly abundant proteins in retentostat.

## Discussion

### Mass transfer limitation acts as a bottleneck in micropollutant degradation at low concentrations and influences the maintenance demand

Growth kinetics of *A. aurescens* TC1 such as a high $q_s^{max}$ and moderate *K*_s_ ~1 µM when compared with copiotrophic microorganisms (10 µM or higher) [48] indicate that atrazine can be effectively biodegraded to concentrations as low as 12 μg L^−1^. Considering that only amine side chains of atrazine are used for energy metabolism, of the eight carbon atoms in atrazine only five will be utilized [9]. Since isotope fractionation provided direct evidence of mass transfer limitation, and since we did not observe evidence of an atrazine transporter that would “boost” intracellular concentrations [42], cell membrane permeation must be diffusion mediated meaning that the concentration inside the cell can never exceed the outside concentration. Indeed, mass transfer limitation through cell membrane makes only 60% of concentrations outside available for metabolism [19]. Hence, at D_low_ and retentostats, the intracellular concentrations are expected to be 18 and 9 µg L^−1^, respectively. It is therefore remarkable that under such a low catabolic energy availability the relative abundances of atrazine hydrolytic proteins were not significantly reduced. Since the enzymes of atrazine hydrolysis were not downregulated — in particular not TrzN, which is the first assimilatory intracellular atrazine degradation enzyme and rather shows a high substrate affinity — this suggests that the enzyme machinery remained fully active and there was no intracellular mechanism to alleviate mass transfer limitation. Degradation of ethylamine and isopropylamine, common moieties of various organic molecules, is known to be much faster than of atrazine [5, 49] and should not be rate limiting. In the absence of transporters, this may reflect a strategy to keep a large stock of intracellular enzymes increasing V_max_ [50] in order to boost diffusion-mediated transport in response to mass transfer limitation at low concentrations. Despite high atrazine degradation, lower yields in chemostats and retentostats than in batch cultivation indicate that a higher fraction of consumed substrate goes to *m*_E_ [31] This may reflect saprophytic competence and survival since K-strategists devote more energy to competitive success and survival than to reproduction [51, 52]. The *m*_E_ here refers to the basal metabolic rate not for dormant cells but in actively metabolizing cells [24, 53]. The *m*_E_ observed in chemostat falls into the upper end of ranges for different aerobic microorganisms (7.6 –116 kJ (C-mol biomass)^−1^ h^−1^) [25], whereas a lower *m*_*E*_ (~37 kJ (C-mol biomass)^−1^ h^−1^) could be observed in retentostat. This reduction in *m*_E_ at near-zero growth rate is in accordance with the concept of lower *m*_E_ associated with the no-growth condition, [54–56] but contrasts with findings of similar *m*_E_ at both high or zero growth rates [26] or even higher *m*_E_ at zero growth rate [57]. When considering all cells to be metabolically active in retentostats, the estimated *m*_E_ corresponds to values that were observed in marine sediment (~1 × 10^−9^ kJ cell^−1^ h^−1^) in situations of high energy turnover [54]. When microorganisms physiologically adapt by synthesizing the same level of enzymes to maintain active degradation at low concentrations [5, 10], they nevertheless add an extra cost to their “energy budget” as there is a structural cost of a gene, especially in the context of transcript turnover and protein decay with time [53, 58]. This is indeed reflected in downregulation of many catabolic and anabolic proteins at D_low_ compared with retentostat: at D_low_ cells were forced to reproduce faster (17 fold shorter *t*_d_ than in retentostat) and the rate of constantly re-synthesizing the whole cell versus replacement of molecules in a cell is supposed to have a first-order control on biosynthesis costs [1]. Moreover, at D_low_ the cell has a high *m*_E_ associated with higher cell volume (Fig. 4) and metabolic rate than in retentostats (Supplementary Table S1) [53, 59].

### Proteome analysis reveals metabolic pathway alterations consistent with survival strategies triggered by energy limitation

To cope with sparse energy availability due to mass transfer limitation, microorganisms may up- and downregulate many proteins indicating a reprogramming of the cellular network to “slow-down” the metabolism. Interestingly, under mass transfer limitation both at D_low_ and in retentostats, many proteins related to the survival under harsh conditions were highly abundant. For example, ATP-dependent glycine betaine/choline transporter (A1R2I8), a protectant against osmotic, thermal, and oxidative stress, which plays a role in the maintenance of intracellular pH [52, 60, 61] was of relatively higher abundance in retentostats and chemostats at D_low_. The same is true for glycine betaine as a stabilizer for proteins and single-stranded nucleic acids [60, 62, 63]. Relative abundance of cupin (A1R5W7), a superfamily of β-barrel structural domains, which plays a role in cell morphogenesis, cell wall structure, and desiccation tolerance was also higher [52, 64]. Further, ClpB, a part of the multi-chaperone system, involved in the recovery of the cell from heat-induced damage were highly expressed [52]. Response to stress was also reflected in the expression of antibiotic resistance proteins [26, 52]. “Derepression of the catabolome,” which has been suggested as a further survival strategy, was not observed in our study [13]. Nevertheless, significantly high abundance of proteins in retentostats also indicate a shift towards amino acids degradation and transport of sugars as alternative energy sources.

### The challenges of sustaining an active metabolism at low concentrations

At low concentrations, depending on the energy availability, microorganisms can exist in three different physiological states: “growth phase,” “maintenance phase”, and “survival phase” [54, 56]. In our study, under partial and full mass transfer limitation, the cell physiology reflected the condition of growth and maintenance phase, respectively. In retentostats, the bioavailable concentration of atrazine in terms of Gibbs free energy yield was much higher than the “biological energy quantum,” the lowest amount of energy that can be conserved by an organism [54, 65]. Nonetheless, cells in retentostats already expressed proteins indicative of a stress-related survival mode and changed their morphology as if responding to extreme energy limitation [1]. Taken together, this indicates that a threshold for long-term atrazine degradation was being approached. It may be hypothesized that below *S*_min_ cells will eventually enter the “survival phase,” and the minimal energy supply caused by slower cell membrane permeation will force microorganisms to further adapt in terms of *m*_E_ sustaining only one component of maintenance, i.e., protein and RNA repair [55]. In the “survival phase,” in turn, maintaining a long-term active degradation will be challenging for organisms with the primary constraint imposed by protein decay with time [53], and this finally may lead to dormancy [55].

However, in nature mass transfer limitation of a specific substrate will not always exert such strong effects since heterotrophic bacterial growth also occurs through the utilization of alternative energy sources such as naturally occurring organic compounds or metabolites produced by other bacteria or microbial necromass. Moreover, growth may also be limited by other factors such as availability of electron acceptor and nutrients. Finally, organisms may have different ways to respond to substrate scarcity, e.g., by active transport, as most recently characterized for the bacterium *Rhizobium* sp. CX-Z [66], or by downregulation of catabolic enzymes [67]. However, despite this complexity, the aim of microbial ecology is often to inform about the management of specific processes. The adaptive response characterized in this study provides an important glimpse to the in situ ecophysiological constraints on specific processes of interest to environmental management that involve low substrate concentrations. The mass transfer limitation observed here might apply to other atrazine-degrading microorganisms as the hydrolysis reaction mechanism by TrZN is the same as that of  another commonly found hydrolytic enzyme, AtzA [11]. Moreover, the ability to observe mass transfer limitation directly through isotope fractionation as accomplished in this study gives first evidence that mass transfer limitation may be the overarching driver which triggers microbial adaptation to low concentrations. Further studies, which take the same approach and focus on adaptive strategies in diverse bacteria from energy-starved environments, can shed light on the role of physiological adaptation and mass transfer limitation that sets a threshold for long-term micropollutant degradation.

## Supplementary information

Supporting Information

Table S3
