# Correction: Defining lower limits of biodegradation: atrazine degradation regulated by mass transfer and maintenance demand in *Arthrobacter aurescens* TC1

**DOI:** 10.1038/s41396-019-0512-y

**Published:** 2019-10-02

**Authors:** Kankana Kundu, Sviatlana Marozava, Benno Ehrl, Juliane Merl-Pham, Christian Griebler, Martin Elsner

**Affiliations:** 1 Institute of Groundwater Ecology, Helmholtz Zentrum München, Ingolstädter Landstraße 1, Neuherberg, Munich, Bavaria D-85764 Germany; 2 Core Facility Proteomics, Helmholtz Zentrum München, Heidemannstr 1, Munich, D-80939 Germany; 3 University of Vienna, Center of Functional Ecology, Division of Limnology, Althanstrasse 14, Vienna, 1090 Austria; 4 Department of Analytical Chemistry and Water Chemistry, Technical University of Munich, Marchioninistrasse 17, Munich, D-81377 Germany

**Keywords:** Environmental microbiology, Environmental sciences

**Correction to: The ISME Journal**

10.1038/s41396-019-0430-z

Since publication of the original article the authors noticed that co-author Christian Griebler’s second affiliation was not included. This has now been added to the HTML and PDF versions of the paper.

Furthermore, Supplementary Table 3 was not uploaded with the rest of the Supplementary Information files. This is now available to view on the HTML of the original article.

The authors would like to apologies for any inconvenience caused.

