# Supplementary material for: Defining lower limits of biodegradation: atrazine degradation regulated by mass transfer and maintenance demand in Arthrobacter aurescens TC1
Source: ISME J. 2019 May 9;13(9):2236–51. doi: 10.1038/s41396-019-0430-z (PMC6776027; doi:10.1038/s41396-019-0430-z)
Supplement: Supplementary file 1 — Supporting Information [file 41396_2019_430_MOESM1_ESM.docx]

**Supporting Information**

Defining lower limits of biodegradation – atrazine degradation regulated by mass transfer and maintenance demand in *Arthrobacter aurescens* TC1

Kankana Kundu, Sviatlana Marozava, Benno Ehrl, Juliane Merl-Pham, Christian Griebler, Martin Elsner

**SI Materials and methods:**

*Parameter estimation*

The two parameter estimates $K_{s}$ and $q_{s}^{max}$ in the substrate consumption rate model (eq.3) were estimated from the experimentally measured $q_{s}$and the residual atrazine concentration ($C_{s})$data by minimizing the Root mean squared error (RMSE) as objective function (eq.7).

(7)

$$\sqrt{\sum\frac{(\vartheta_{exp}-{\vartheta_{sim})}^{2}}{N}}$$

The “brute force” optimization method was used to find the global minimum of the objective function to compute the objective function’s value at each point of a multidimensional grid of points, to obtain the global minimum of the function. This multidimensional grid contained ranges of $q_{s}^{max}$ (0.001 to 10) and $K_{s}$ (10 to 1000) with linear grid space of 0.005 and 1, respectively. Thereafter, the result of “brute force” minimization was fed as initial guess to obtain a more precise (local) minimum using the downhill simplex algorithm [1]. A similar approach was taken to estimate $Y_{x/s}^{max}$and $m_{s}$ for chemostat (equation 4) and retentostat cultivations (equation 5).

*Model analysis*

The RMSE as a measure of the discrepancy between the experimental data and model prediction is used to indicate an accuracy of the model to describe the kinetics of chemostat and retentostat culture. A small RMSE indicates a close fit of the model to the data. Furthermore, to check the functional relation and reliability of estimated parameters, practical identifiability was examined using the collinearity analysis over the complete parameter space $\mathfrak{R}$. If a vector $\beta$ exists among columns $\mathfrak{R}_{k}$= 1, 2, ..., m, of $\mathfrak{R}$ such that $\parallel\beta\parallel\neq0$ and $\mathfrak{R}\beta\approx0$, they are near collinear. This collinearity was tested by inspecting the smallest eigenvalue $\lambda_{m}$ of the normalised sensitivity matrix, $\overset{̃}{\mathfrak{R}}$ [2]. The collinearity index, $\gamma$ is calculated as

(8)

$$\gamma=\frac{1}{\min_{\parallel\beta\parallel=0}\parallel\overset{̃}{\mathfrak{R}}\beta\parallel}=\frac{1}{\sqrt{\lambda_{m}}}$$

Parameter subsets below the collinearity index of 5 are considered as identifiable and above 20 are considered as non-identifiable [2]. The model implementation, fitting parameter estimations and model analysis was performed using Python and employing the built-in functions in scientific libraries NumPy and SciPy [3].

*HPLC analysis*

For substrate and metabolite analysis, samples were filter-sterilized and stored at ${-20}^{\circ}$C before HPLC analysis. Atrazine and 2-hydroxyatrazine concentrations were measured using a Prominence HPLC system (Shimadzu Corp., Japan) together with a 100 x 4.6 mm Kinetex 5 µ Biphenyl 100 Å column equipped with a SecurityGuard ULTRA Biphenyl cartridge (both Phenomenex Inc., USA). Peak separation was achieved by 1 mL/min isocratic flow of 51 % 5 mM KH_2_PO_4_ buffer pH 7 and 49 % methanol for 9 min. The compounds were detected by UV absorbance at 222 nm and the peaks were quantified using LabSolutions V 5.71 SP2 (Shimadzu Corp., Japan).

*Cell concentration determination by Flow Cytometry and cell morphology*

For flow cytometry analysis, the sample aliquots (mostly 10 $\mu L)$were stained with 1$\mu L$ SYBR Green (1000x working solution, Invitrogen-Thermo Scientific, MA, USA) and 1$\mu L$ Propidium iodide (1mg mL^-1^, Invitrogen-Thermo Scientific, MA, USA). Two analysis, one for SYBR Green I stained and one for propidium iodide stained cells, were performed separately (both in biological duplicates and technical duplicates) on a Cytomics FC 500 flow cytometer (Beckmann Coulter, Germany) equipped with a 488 nm (40 mW) and a 638 nm (25 mW) laser. The FCM set-up was as follows: SYBR Green I stained samples: discriminator FL1 (green fluorescence) /0, forward scatter 178 V/ gain 2.0, side scatter 624 V/ gain 2.0, FL1 397 V/ gain 1.0, and FL3 (red fluorescence) 572 V/ gain 1.0. Propidium iodide stained samples: discriminator FL3 /1, forward scatter 745 V/ gain 1.0, side scatter 693 V/ gain 2.0, FL1 350/ gain 1.0, FL2 (yellow fluorescence) 527 V/ gain 1.0, and FL3 517 V/ gain 1.0. As an internal standard, fluorescence beads ($\mathbf{Tucount}^{TM}$ Absolute Counting Tubes, BD Bioscience, USA) of a known concentration dissolved in freshly prepared and 0.22 $\mu$m filtered PBS were added to each sample. Data were analyzed with the CXP software (version 2.2; Beckman Coulter, Germany). The following formula was used for the calculation of total cell numbers

$$Cells \left[ {mL}^{-1} \right]=\frac{events\times reference/tube\times}{reference limit \left( 200 \right)\times total sample volume (1mL)}\times dilution \times fix factor (1.1)$$

For cell morphology in chemostats and retentostats, the cells were analyzed on agar glass slides by light microscopy with an Axioscope 2 Plus microscope (Carl Zeiss AG, Germany). For imaging, pictures were taken with the digital camera AxioCam HRm (Carl Zeiss AG, Germany) and the software AxioVision (Version 4.8.2; Carl Zeiss AG, Germany). The same software was used to measure the diameter/length of the cells. At least 45 randomly chosen cells were analyzed for the samples.

*Thermodynamic calculation*

Atrazine is first hydrolyzed by TrzN to hydroxyatrazine. Then, ethyl amine is cleaved off by hydrolysis with AtzB and AtzC catalyzes the hydrolysis to cyanuric acid and cleaves of isopropylamine. Cyanuric acid is discarded, while the short chain amines serve as a further source of energy and growth [4]. Since the initial hydrolysis is energy-neutral, thermodynamic calculations were based on conversion of the short chain amines. Therefore, the Gibbs free energy released during substrate catabolism to CO_2_ (${\Delta_{R}G^{0'}}_{\mathrm{cat}}$) and for growth (${\Delta_{R}G^{0'}}_{\mathrm{ana}}$) were calculated using the following stoichiometric equations. Free energy values under standard conditions ($\Delta_{R}G^{0'}$) were calculated from published $\Delta_{f}G^{0'}$ values [5, 6].

$$C_{2}H_{5}NH_{3}+C_{3}H_{7}NH_{3}+7.5O_{2}\underset{\to}{}5HCO_{3}^{-}+5H^{+}+2NH_{4}^{+} {\Delta_{R}G^{0'}}_{\mathrm{cat}}= -3338 kJ.{mol}^{-1}$$

$$C_{2}H_{5}NH_{3}+C_{3}H_{7}NH_{3}+4O_{2}\underset{\to}{}2.2HCO_{3}^{-}+5H^{+}+1.44NH_{4}^{+}+2.8CH_{1.8}O_{0.5}N_{0.2}$$

${\Delta_{R}G^{0'}}_{\mathrm{ana}} = -1838 kJ.{mol}^{-1}$

*Isotope fractionation analysis*

Atrazine was extracted with dichloromethane (10 % of the sample volume, three times). The GC-IRMS system consisted of a TRACE GC ultra gas chromatograph (GC; Thermo Fisher Scientific, Milan, Italy) linked to a Finnigan MAT 253 isotope ratio mass spectrometer (IRMS) (Thermo Fisher Scientific, Germany) by a Finnigan GC Combustion III Interface (Thermo Fisher Scientific, Germany). The samples were injected using a GC Pal autosampler (CTC, Switzerland) onto a 60-m DB-5 (60 m × 0.25 mm; 1 μm film; Restek GmbH, Germany) analytical column. An on-column liner (custom made by a glassblower) was pressed directly onto a CS-fused-silica-methyl-sil retention gap (3 m × 0.53 mm inner diameter) (Chromatographie Service GmbH, Germany). The GC oven started at 35 °C (hold 30 s), ramp 5 °C/min to 80 °C to ensure complete solvent evaporation during the transfer of the sample from the retention gap to analytical column. This was followed by a temperature ramp of 100 °C/min to 160 °C, a ramp of 10 °C/min to 220 °C, then a ramp 2 °C/min up to 250 °C. The initial injector temperature at the Optic 3 was set to 40 °C, 300 s hold, then ramped to 250 °C at 2 °C/s. The initial column flow was set to 0.3 mL/min (hold 120 s), then ramped to 1.4 mL/min within 120 s so that a flow of 1.4 mL/min was established before the GC temperature was raised [7].

Isotopic enrichment factors were determined by determining the difference of the isotope ratios between the inflow and the outflow: Alternating measurements of the inflow and the bioreactor multiple times allowed the determination of the difference without additional uncertainty of the instrument.

*Protein Extraction*

Samples were centrifuged at 8,000 rpm, $4^{\circ}$C for 20 min in Avanti J-E centrifuge (Beckman Coulter Inc., USA). Then cells were washed once with 1×phosphate buffered saline (PBS) consisting of (per liter) NaCl (8 g), KCl (0.2 g), KH_2_PO_4_ (0.24 g), and Na_2_HPO_4_ (1.44 g). Washed cells were centrifuged again at 15,300 × g for 5 min at $4^{\circ}$C. The cell pellet was dissolved in lysis buffer (9M urea, 2M thiourea, 4% CHAPS, 1% DTT) containing proteinase inhibitor (Complete Mini. EDTA-free (Roche, Germany)) and incubated 30 min at room temperature. Protein extraction was performed via sonication: sonication was applied twice for 1 min (0.3 s per pulse, 30% duty) (ultrasonic processor UP50H, Hielscher Ultrasonics, Germany) with sample cooling on ice between the rounds. DNA/RNase digestion was performed via adding appropriate amount of Nuclease-Mix (GE Healthcare, Germany) to the supernatant and incubating samples for 30 min at room temperature. Protein concentration was determined using the Bradford protein assay (Bio-Rad) with bovine serum albumin as the standard [8].

**SI Figures and Tables:**

**
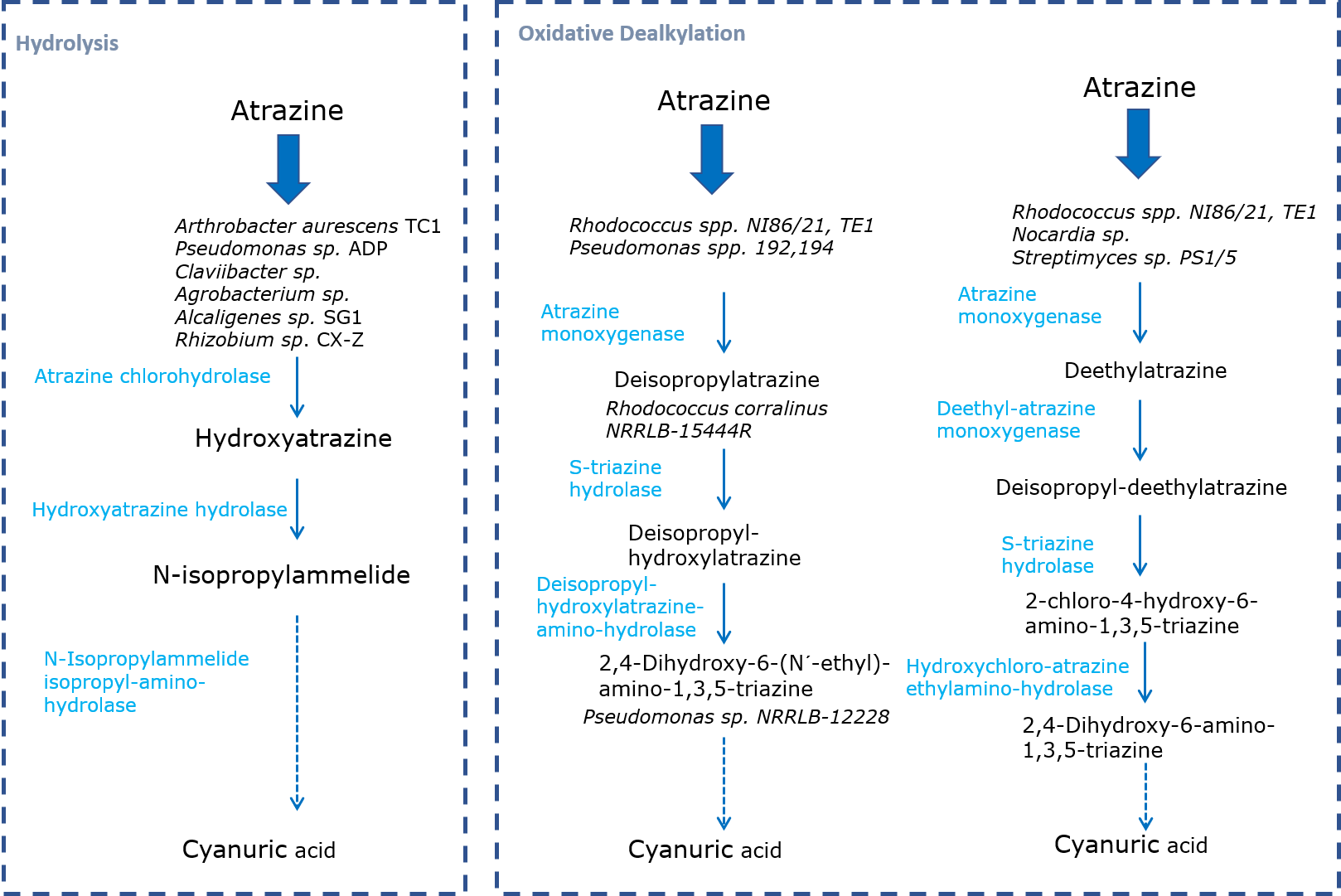
**

Fig. S1: Degradation of atrazine involves two pathways [9–11]. The microorganisms which can initiate the degradation or carry out later steps are indicated.

**
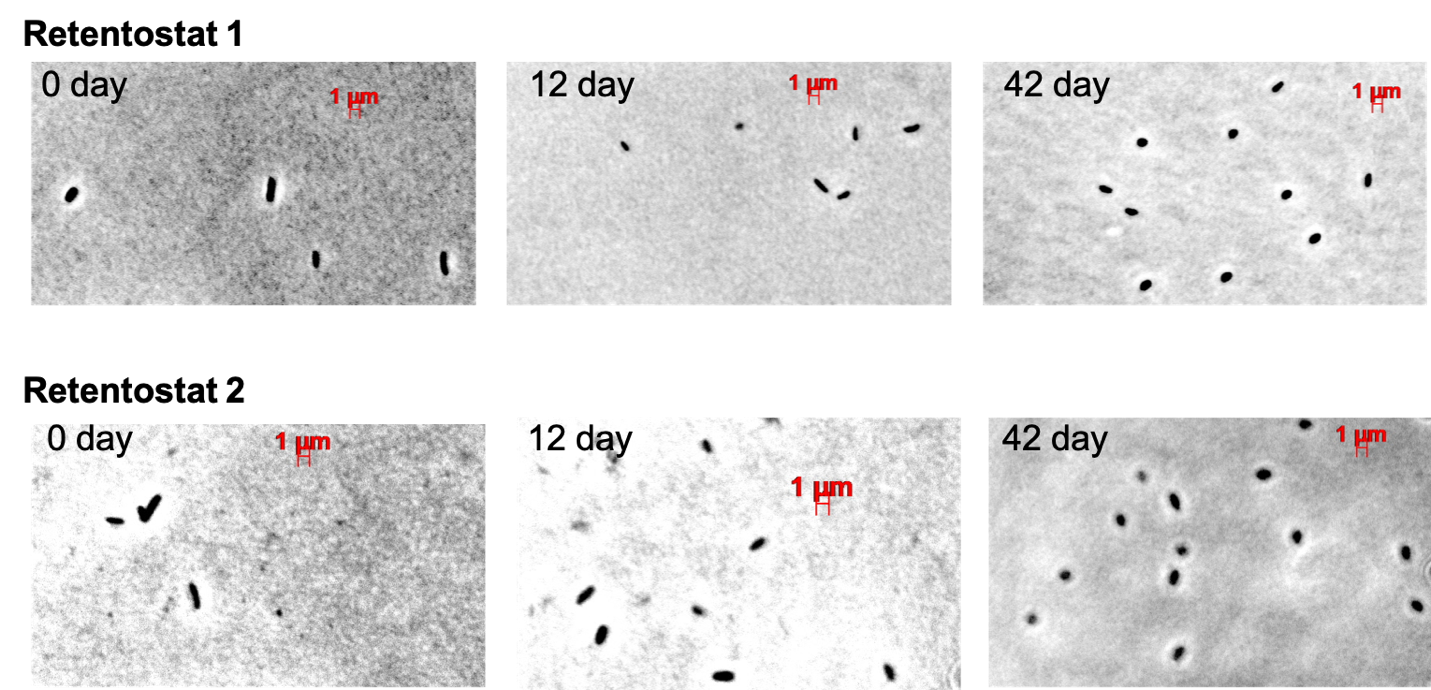
**

Figure S2: Changes in cell morphology in time in water samples from the retentostats.


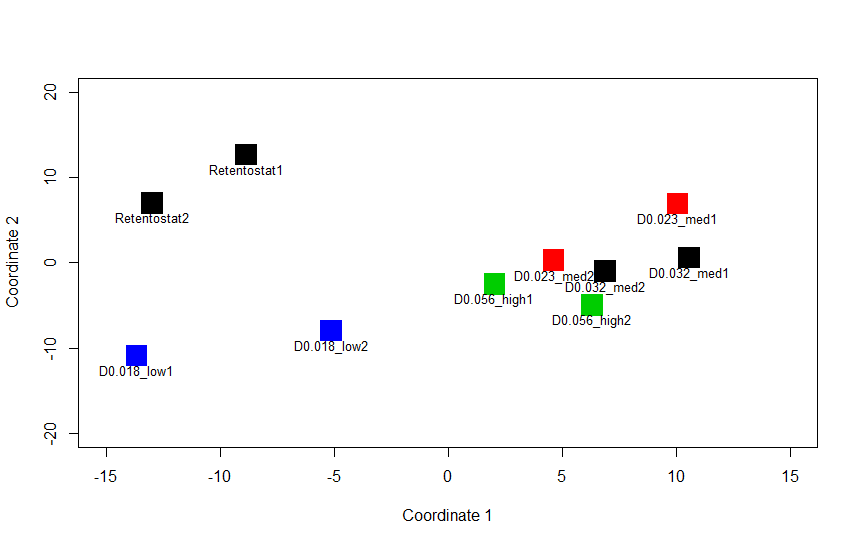


Figure S3: Non-parametric clustering (NMDS) of all conditions used for proteomics analysis.


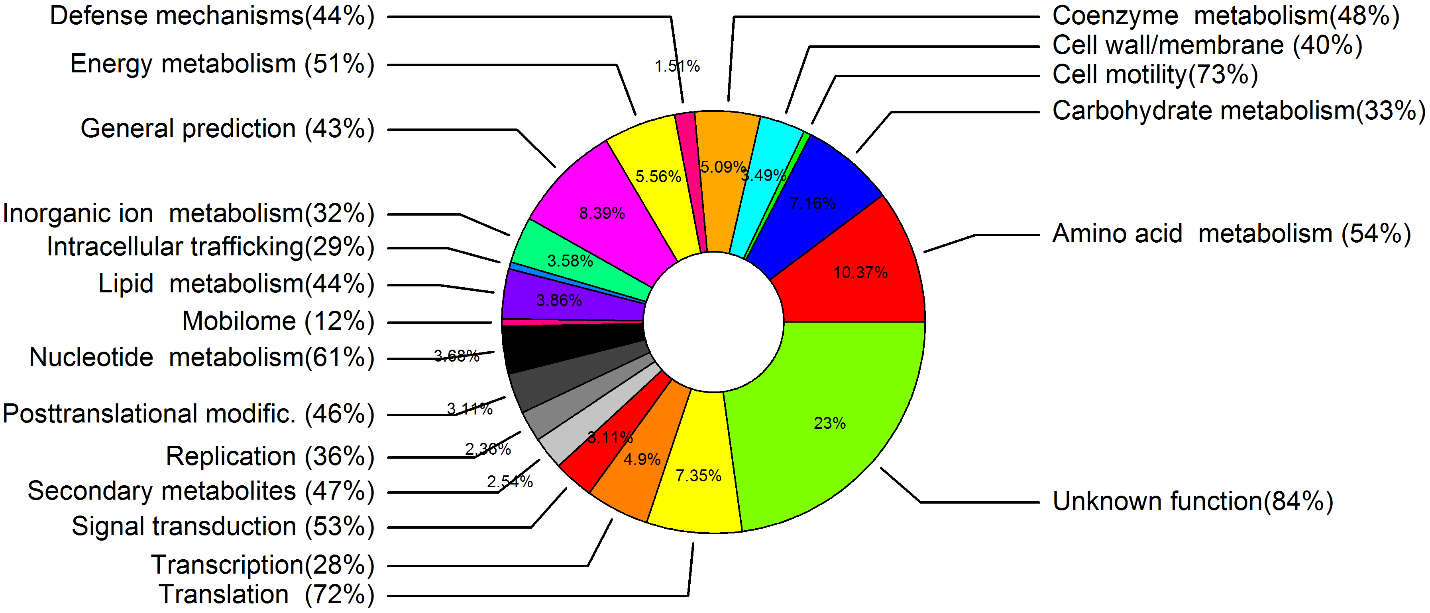


Figure S4: Assignment of total identified proteins (1735) to clusters of orthologous groups (COG). Number in brackets next to the categories indicate the percentage of proteins from each category in respect to be predicted . Most of the detected categories had at least 40% proteins from predicted to be in particular category. [12]

Table S1: Distribution of energy flux to maintenance during chemostat and retentostat cultivation. D-Dilution rate, HRT-Hydraulic retention time.

|  |  | **Chemostat** |  |
| --- | --- | --- | --- |
| D (h^-1^) | HRT (h) | Atrazine supply rate mg h^-1^ | % of flux used for maintenance |
| 0.006 | 166 | 0.36 | 81 |
| 0.009 | 111 | 0.54 | 54 |
| 0.018 | 55 | 1.08 | 29 |
| 0.023 | 43 | 1.45 | 23 |
| 0.032 | 31 | 2.01 | 16 |
| 0.048 | 20 | 3.02 | 13 |
| 0.056 | 17 | 3.52 | 10. |
| 0.068 | 14 | 4.28  **Retentostat** | 9. |
| D (h^-1^) | Time (h) | Atrazine supply rate mg h^-1^ | % of flux used for maintenance |
| 0.02 | 0 | 1.29 | 26 |
| 0.02 | 216 | 1.29 | 73. |
| 0.02 | 528 | 1.29 | 84 |
| 0.02 | 912 | 1.29 | 97 |

Table S2: Total number of proteins detected in biological replicates under different conditions in chemostats and retentostats.

| **Chemostat** | | | |
| --- | --- | --- | --- |
| D (h^-1^) | Viable cells (# mL^-1^) | Sampling volume  (mL) | Proteins quantified |
| 0.018 |  |  |  |
| *-Replicate 1* | 2.20× 10^7^ | 200 | 1665 |
| *-Replicate 2* | 2.76× 10^7^ | 200 | 1675 |
| 0.023 |  |  |  |
| *-Replicate 1* | 2.17 × 10^7^ | 200 | 1698 |
| *-Replicate 2* | 2.44 × 10^7^ | 200 | 1702 |
| 0.032 |  |  |  |
| -*Replicate 1* | 2.22× 10^7^ | 200 | 1702 |
| *-Replicate 2* | 2.55× 10^7^ | 200 | 1699 |
| 0.056 |  |  |  |
| -*Replicate 1* | 2.62× 10^7^ | 200 | 1682 |
| *-Replicate 2* | 2.74× 10^7^ | 200 | 1697 |
| **Retentostat** | | | |
| D (h^-1^) | Viable cells (# mL^-1^) | Sampling volume  (mL) | Proteins quantified |
| 0.02 |  |  |  |
| *-Replicate 1* | 2.75× 10^8^ | 20 | 1707 |
| *-Replicate 2* | 2.12× 10^8^ | 20 | 1691 |

Table S4 Differentially abundant proteins specific to the retentostats compared to both Chemostats at D_high_ +D_medium_  and D 0.018 h^-1^. These proteins were shared in between pairwise comparisons- Retentostat vs Chemostats at D_high_ +D_medium_; and Retentostat vs D 0.018 h^-1^ . Proteins in pairwise comparison with a P value of <0.05 with a cut off log fold change (LFC) of log_2_(2.5) were considered to be significantly differentially abundant [13]. P values were adjusted by Benjamini−Hochberg based false discovery rate correction method.

| **Upregulated proteins** | |  |  |  |  |
| --- | --- | --- | --- | --- | --- |
| **UniProt ID** | **Description** | **LFC (RET-CHEM)** | **Adj.P.value (Ret-CHEM)** | **LFC (RET-D0.018)** | **Adj.P.value (Ret-D0.018)** |
|  | **General function prediction only** |  |  |  |  |
| A1RCX5 | Hydroxyatrazine hydrolase | 1.479 | 0.025 | 2.372 | 0.019 |
| A1R6U9 | Uncharacterized protein | 1.392 | 0.047 | 3.680 | 0.019 |
| A1R4H3 | 'helix-loop-helix' dimerization domain signature protein | 2.921 | 0.004 | 5.860 | 0.017 |
|  | **Amino acid transport and metabolism** |  |  |  |  |
| A1R5T1 | Glutamate synthase large subunit | 1.667 | 0.013 | 2.612 | 0.021 |
| A1R116 | Putative peptidase family S51 protein | 4.782 | 0.004 | 3.433 | 0.013 |
| A1R3N4 | Putative agmatinase (SpeB) | 2.246 | 0.008 | 1.709 | 0.043 |
| A1R2Y6 | Putative amino acid decarboxylase, pyridoxal-dependent protein | 1.603 | 0.014 | 5.430 | 0.011 |
| A1RAE9 | Aminotransferase, class V protein | 1.392 | 0.038 | 3.220 | 0.011 |
|  | **Carbohydrate transport and metabolism** |  |  |  |  |
| A1R3D0 | Phosphoglycerate mutase family protein | 2.272 | 0.010 | 2.803 | 0.011 |
| A1R438 | Putative sugar ABC transporter, ATP-binding protein | 7.204 | 0.003 | 2.394 | 0.021 |
| A1R671 | Putative ABC-type sugar transport system | 6.030 | 0.012 | 2.646 | 0.022 |
|  | **Cell wall/membrane/envelope biogenesis** |  |  |  |  |
| A1R6D5 | Uncharacterized protein | 3.893 | 0.002 | 7.619 | 0.011 |
| A1R6N1 | Putative UDP-glucose 4-epimerase | 1.680 | 0.017 | 4.614 | 0.044 |
|  | **Coenzyme transport and metabolism** |  |  |  |  |
| A1R6A2 | Riboflavin biosynthesis protein RibD | 2.338 | 0.005 | 2.965 | 0.022 |
|  | **Defense mechanisms** |  |  |  |  |
| A1RC79 | Putative ABC transporter, ATP-binding protein | 1.692 | 0.005 | 2.018 | 0.031 |
|  | **Energy production and conversion** |  |  |  |  |
| A1R307 | Pyridine nucleotide-disulphide oxidoreductase domain protein | 2.982 | 0.003 | 2.287 | 0.021 |
| A1R4L6 | Dihydrolipoyl dehydrogenase | 1.649 | 0.009 | 1.805 | 0.019 |
| A1R181 | Mitomycin radical oxidase | 1.946 | 0.010 | 1.887 | 0.048 |
|  | **Function unknown** |  |  |  |  |
| A1RA42 | Putative cupin domain protein | 2.189 | 0.029 | 1.958 | 0.019 |
| A1R575 | Uncharacterized protein | 2.420 | 0.042 | 1.892 | 0.040 |
|  | **Lipid transport and metabolism** |  |  |  |  |
| A1R6M5 | Putative acyl-CoA dehydrogenase | 1.540 | 0.044 | 3.271 | 0.014 |
|  | **Nucleotide transport and metabolism** |  |  |  |  |
| A1R6Z7 | Aspartate carbamoyltransferase | 1.815 | 0.014 | 2.996 | 0.011 |
|  | **Posttranslational modification, protein turnover, chaperones** |  |  |  |  |
| A1R6Q7 | Proteasome subunit beta | 2.162 | 0.020 | 4.260 | 0.011 |
|  | **Replication, recombination and repair** |  |  |  |  |
| A1R2K7 | Recombination protein RecR | 3.114 | 0.025 | 3.672 | 0.048 |
| A1R6B6 | Putative 5'-3' exonuclease | 2.179 | 0.006 | 2.504 | 0.017 |
|  | **Secondary metabolites biosynthesis, transport and catabolism** |  |  |  |  |
| A1R2Y4 | Putative glyoxalase family protein | 1.896 | 0.025 | 2.515 | 0.031 |
|  | **Transcription** |  |  |  |  |
| A1R201 | RNA polymerase sigma factor | 4.449 | 0.012 | 4.365 | 0.046 |
| A1RDF3 | Transcriptional regulator, AraC family | 4.566 | 0.020 | 4.984 | 0.029 |
|  | **Unknown** |  |  |  |  |
| A1R823 | FG-GAP repeat domain protein | 4.809 | 0.001 | 5.085 | 0.003 |
| A1R820 | Uncharacterized protein | 2.616 | 0.001 | 3.107 | 0.005 |
| A1RD77 | Uncharacterized protein | 3.765 | 0.003 | 4.211 | 0.011 |
| A1RAN7 | Uncharacterized protein | 3.424 | 0.006 | 3.598 | 0.022 |
| A1RCC2 | T Putative Lipoprotein | 2.518 | 0.004 | 3.036 | 0.011 |
| A1RD03 | Uncharacterized protein | 1.374 | 0.049 | 2.010 | 0.047 |
| A1R9K2 | Putative membrane protein | 3.803 | 0.013 | 4.927 | 0.021 |
| A1R1J0 | Uncharacterized protein | 1.374 | 0.044 | 2.808 | 0.016 |
| A1R5M0 | Uncharacterized protein | 3.506 | 0.003 | 3.413 | 0.016 |
| A1R4G5 | Putative transcriptional regulator, LuxR family | 1.670 | 0.025 | 3.956 | 0.008 |
| A1R3M2 | FG-GAP repeat domain protein | 6.482 | 0.001 | 7.649 | 0.003 |
| A1RD74 | Uncharacterized protein | 5.378 | 0.004 | 7.058 | 0.011 |
| A1R3T7 | CBS domains protein | 5.794 | 0.001 | 3.792 | 0.022 |
| A1R657 | ANTAR domain protein | 4.734 | 0.028 | 6.170 | 0.039 |
| A1RCI2 | GAF domain protein | 3.315 | 0.026 | 5.033 | 0.022 |
| A1RA33 | Putative lipoprotein | 5.380 | 0.003 | 4.699 | 0.019 |
| A1R1L8 | Putative lipoprotein | 4.204 | 0.004 | 3.470 | 0.028 |
| A1R8E2 | Uncharacterized protein | 3.688 | 0.003 | 3.187 | 0.021 |
| A1R7Z2 | Uncharacterized protein | 3.610 | 0.001 | 3.183 | 0.005 |
| A1R7H9 | Uncharacterized protein | 4.334 | 0.002 | 4.531 | 0.011 |
| A1R9A4 | Uncharacterized protein | 1.913 | 0.017 | 2.483 | 0.024 |
| A1R6N3 | Putative Acetyltransferase protein | 4.219 | 0.026 | 6.129 | 0.024 |
| **Downregulated proteins** | |  |  |  |  |
|  | **Amino acid transport and metabolism** |  |  |  |  |
| A1R858 | Chorismate mutase | -2.316 | 0.020 | -3.627 | 0.017 |
| A1R7W8 | Homoserine kinase | -1.610 | 0.010 | -1.822 | 0.024 |
|  | **Carbohydrate transport and metabolism** |  |  |  |  |
| A1R8S2 | sn-glycerol-3-phosphate-binding protein | -1.845 | 0.008 | -1.796 | 0.035 |
|  | **Cell wall/membrane/envelope biogenesis** |  |  |  |  |
| A1R683 | Putative dehydrogenase | -1.842 | 0.042 | -2.531 | 0.047 |
| A1R9E6 | Glucose-1-phosphate thymidylyltransferase | -3.886 | 0.001 | -2.850 | 0.014 |
|  | **Coenzyme transport and metabolism** |  |  |  |  |
| A1R8Y8 | Dihydrofolate reductase | -6.735 | 0.002 | -5.663 | 0.027 |
|  | **Energy production and conversion** |  |  |  |  |
| A1R110 | Putative nitroreductase family protein | -3.557 | 0.008 | -3.628 | 0.029 |
|  | **Function unknown** |  |  |  |  |
| A1R633 | Uncharacterized protein | -3.251 | 0.004 | -2.833 | 0.029 |
|  | **General function prediction only** |  |  |  |  |
| A1R4N7 | Putative methanol dehydrogenase regulatory protein | -4.145 | 0.001 | -3.072 | 0.018 |
| A1R221 | Putative fasciclin domain protein | -2.818 | 0.005 | -3.199 | 0.017 |
| A1RAV7 | Putative major facilitator superfamily (MFS) transporter | -2.472 | 0.005 | -3.560 | 0.011 |
|  | **Unknown** |  |  |  |  |
| A1RDK4 | Putative Pyridoxamine 5'-phosphate oxidase | -5.237 | 0.004 | -3.967 | 0.036 |
| A1R5K1 | Putative glyoxalase family protein | -6.495 | 0.001 | -4.794 | 0.014 |
| A1R402 | Putative transcriptional regulator, LuxR family | -1.539 | 0.048 | -2.279 | 0.044 |

Table S5 Differentially abundant proteins specific to D 0.018 h^-1^ compared to both Chemostats at D_high_ +D_medium_  and retentostats. These proteins were shared in between pairwise comparisons- D 0.018 h^-1^ vs Chemostats at D_high_ +D_medium_; and D 0.018 h^-1^ vs Retentostat. Proteins in pairwise comparision with a P value of <0.05 with a cut off log fold change (LFC) of log_2_(2.5) were considered to be differentially abundant [13]. P values were adjusted by Benjamini−Hochberg based false discovery rate correction method.

| **Upregulated proteins** | | **LFC (D0.018-CHEM)** | **Adj.P.value (D0.018-CHEM)** | **LFC (D0.018-RET)** | **Adj.P.value (D0.018-RET)** |
| --- | --- | --- | --- | --- | --- |
| **UniProt ID** | **Description** |  |  |  |  |
|  | **Cell wall/membrane/envelope biogenesis** |  |  |  |  |
| A1R7B0 | Putative NAD dependent epimerase/dehydratase family protein | 1.905 | 0.0300 | 2.9054 | 0.026 |
|  | **General function prediction only** |  |  |  |  |
| A1R360 | Putative 3-oxoacyl-[acyl-carrier protein] reductase | 3.481 | 0.0051 | 2.7096 | 0.0240 |
|  | **Unknown** |  |  |  |  |
| A1R648 | Putative lipoprotein | 2.372 | 0.0397 | 4.2806 | 0.0220 |
| **Downregulated proteins** | |  |  |  |  |
|  | **Amino acid transport and metabolism** |  |  |  |  |
| A1RDE8 | Putative amino acid permease | -2.296 | 0.0209 | -2.8339 | 0.0321 |
| A1RBV1 | Putative 4-hydroxyphenylpyruvate dioxygenase | -2.156 | 0.0335 | -3.6215 | 0.0224 |
| A1R7M0 | Acetolactate synthase, small subunit | -1.910 | 0.0302 | -2.4491 | 0.0482 |
|  | **Carbohydrate transport and metabolism** |  |  |  |  |
| A1R5H5 | Polyphosphate glucokinase | -2.912 | 0.0293 | -3.9031 | 0.0392 |
| A1R8N5 | Putative sugar kinase | -3.903 | 0.0323 | -5.5106 | 0.0301 |
| A1R801 | Putative histidinol-phosphate phosphatase, inositol monophosphatase family | -1.668 | 0.0243 | -2.1570 | 0.0352 |
| A1RAC8 | Putative thuA-like protein | -3.270 | 0.0050 | -3.3272 | 0.0114 |
|  | **Coenzyme transport and metabolism** |  |  |  |  |
| A1R155 | GTP cyclohydrolase 1 | -2.880 | 0.0300 | -4.977 | 0.019 |
| A1R8A0 | Delta-aminolevulinic acid dehydratase | -1.493 | 0.0335 | -2.1196 | 0.0407 |
| A1RBS5 | Riboflavin biosynthesis protein RibD | -2.391 | 0.0300 | -4.4450 | 0.0158 |
| A1R984 | Putative cbiX family protein | -1.931 | 0.0209 | -2.2218 | 0.0404 |
|  | **Energy production and conversion** |  |  |  |  |
| A1R6X8 | Glycerol-3-phosphate dehydrogenase | -1.555 | 0.0377 | -2.4362 | 0.0331 |
|  | **Function unknown** |  |  |  |  |
| A1R914 | Putative MOSC domain protein | -3.497 | 0.0124 | -4.7540 | 0.0114 |
| A1R6L5 | Uncharacterized protein | -4.278 | 0.0125 | -4.6446 | 0.0212 |
|  | **General function prediction only** |  |  |  |  |
| A1R8P1 | Oxidoreductase, aldo/keto reductase family | -1.895 | 0.0124 | -2.1445 | 0.0187 |
| A1R4H3 | 'helix-loop-helix' dimerization domain signature protein | -3.440 | 0.0300 | -5.8600 | 0.0172 |
| A1RC65 | Putative phenazine biosynthesis protein, PhzF family | -3.658 | 0.0066 | -2.9028 | 0.0307 |
| A1R677 | Putative myo-inositol 2-dehydrogenase | -7.972 | 0.0172 | -7.9224 | 0.0215 |
|  | **Inorganic ion transport and metabolism** |  |  |  |  |
| A1RCN9 | Putative cobalt-zinc-cadmium efflux permease | -8.133 | 0.0081 | -7.1482 | 0.0212 |
|  | **Lipid transport and metabolism** |  |  |  |  |
| A1R4Z8 | 1-deoxy-D-xylulose 5-phosphate reductoisomerase | -1.700 | 0.0284 | -2.2362 | 0.0404 |
| A1R6M5 | Putative acyl-CoA dehydrogenase | -1.731 | 0.0284 | -3.2709 | 0.0143 |
| A1R4Y7 | 3-oxoacid CoA-transferase, B subunit | -1.400 | 0.0209 | -1.8707 | 0.0245 |
|  | **Nucleotide transport and metabolism** |  |  |  |  |
| A1RAF3 | Uncharacterized protein | -6.075 | 0.0050 | -4.9061 | 0.0158 |
| A1R3L2 | MutT/nudix family protein | -7.716 | 0.0050 | -5.310 | 0.027 |
| A1R4L5 | Purine nucleoside phosphorylase | -2.221 | 0.0209 | -2.7794 | 0.0301 |
|  | **Posttranslational modification, protein turnover, chaperones** |  |  |  |  |
| A1R6Q7 | Proteasome subunit beta | -2.098 | 0.0233 | -4.2596 | 0.0114 |
| A1R9S3 | Putative Tat (Twin-arginine translocation) domain protein | -3.239 | 0.0155 | -3.9277 | 0.0192 |
|  | **Secondary metabolites biosynthesis, transport and catabolism** |  |  |  |  |
| A1R1D7 | 2,5-diketo-D-gluconic acid reductase | -1.493 | 0.0300 | -1.9607 | 0.0426 |
| A1R6S8 | Putative chalcone synthase (Naringenin-chalcone synthase) | -1.579 | 0.0209 | -1.8740 | 0.0380 |
|  | **Signal transduction mechanisms** |  |  |  |  |
| A1R0U8 | Putative serine/threonine kinase domain protein | -2.296 | 0.0125 | -2.0520 | 0.0416 |
| A1R872 | Uncharacterized protein | -2.417 | 0.0074 | -2.0358 | 0.0278 |
|  | **Transcription** |  |  |  |  |
| A1R224 | Putative transcriptional regulator, IclR family | -3.427 | 0.0125 | -3.985 | 0.019 |
| A1R0W8 | Transcriptional regulator, GntR family | -2.442 | 0.0209 | -2.9033 | 0.0388 |
|  | **Translation, ribosomal structure and biogenesis** |  |  |  |  |
| A1R5G8 | Pseudouridine synthase | -4.951 | 0.0167 | -5.6336 | 0.0245 |
|  | **Unknown** |  |  |  |  |
| A1R856 | Uncharacterized protein | -2.604 | 0.0197 | -3.7151 | 0.0187 |
| A1R7M4 | Uncharacterized protein | -2.670 | 0.0243 | -3.8284 | 0.0245 |
| A1R2I2 | Uncharacterized protein | -2.813 | 0.0081 | -2.6619 | 0.0219 |
| A1RBE8 | Uncharacterized protein | -2.823 | 0.0186 | -3.2423 | 0.0245 |
| A1R1J0 | Uncharacterized protein | -1.434 | 0.0332 | -2.8077 | 0.0158 |
| A1R4G5 | Putative transcriptional regulator, LuxR family | -2.286 | 0.0125 | -3.9560 | 0.0084 |
| A1RAC6 | Uncharacterized protein | -2.043 | 0.0051 | -2.2908 | 0.0114 |
| A1R9Q2 | Uncharacterized protein | -5.041 | 0.0120 | -3.8679 | 0.0426 |
| A1RDG7 | Uncharacterized protein | -1.480 | 0.0306 | -2.2296 | 0.0286 |
| A1R9P0 | Uncharacterized protein | -3.109 | 0.0493 | -6.2654 | 0.0212 |
| A1R628 | Uncharacterized protein | -1.870 | 0.0209 | -2.0067 | 0.0489 |

Table S6 Differentially abundant proteins specific to chemostats at D_high_ +D_medium_  compared to both at D 0.018 h^-1^ and retentostats. These proteins were shared in between pairwise comparisons- Chemostats at D_high_ +D_medium_ vs Retentostat; and Chemostats at D_high_ +D_medium_ vs D 0.018 h^-1^. Proteins in pairwise comparision with a P value of <=0.05 with a cut off log fold change (LFC) of log2(2.5) were considered to be differentially abundant [13]. P values were adjusted by Benjamini−Hochberg based false discovery rate correction method.

| **Upregulated proteins** | |  |  |  |  |
| --- | --- | --- | --- | --- | --- |
| **UniProt ID** | **Description** | **LFC (CHEM-RET)** | **Adj.P.value (CHEM-RET)** | **LFC (CHEM-D0.018)** | **Adj.P.value (CHEM-D0.018)** |
|  | **Energy production and conversion** |  |  |  |  |
| A1R953 | Aldehyde dehydrogenase (NAD) family protein | 2.717 | 0.006 | 2.757 | 0.005 |
| A1R6U3 | Cytochrome c oxidase, subunit I | 3.154 | 0.004 | 1.432 | 0.028 |
| A1R5K3 | Putative 2-oxoglutarate dehydrogenase, E2 component, dihydrolipoamide succinyltransferase | 1.655 | 0.005 | 1.919 | 0.019 |
| A1R7E9 | Pyruvate dehydrogenase E1 component | 2.439 | 0.030 | 1.758 | 0.014 |
|  | **Amino acid transport and metabolism** |  |  |  |  |
| A1RDQ3 | Amino acid permease, purative | 2.376 | 0.002 | 1.875 | 0.020 |
| A1R950 | Putative amino acid permease | 3.227 | 0.004 | 2.357 | 0.020 |
| A1RAS9 | Aminotransferase, class IV family | 2.549 | 0.012 | 2.067 | 0.018 |
| A1R8D7 | Glutamate permease | 1.872 | 0.008 | 1.637 | 0.012 |
| A1RDP1 | Alanine dehydrogenase | 1.570 | 0.010 | 1.344 | 0.030 |
| A1R8D9 | Succinyl-diaminopimelate desuccinylase | 6.274 | 0.012 | 2.517 | 0.028 |
| A1R6L2 | Aminopeptidase N | 1.438 | 0.005 | 3.104 | 0.007 |
| A1R1F3 | Putative cyclase family protein | 3.308 | 0.047 | 2.326 | 0.008 |
| A1R1F2 | Putative acetolactate synthase, large subunit | 4.276 | 0.036 | 2.227 | 0.006 |
| A1R2U0 | Fe(3+) ions import ATP-binding protein FbpC | 3.081 | 0.019 | 4.202 | 0.012 |
| A1R588 | Acetylornithine aminotransferase | 1.636 | 0.016 | 2.384 | 0.012 |
| A1RAQ3 | Prephenate dehydratase | 2.308 | 0.016 | 3.913 | 0.021 |
|  | **Carbohydrate transport and metabolism** |  |  |  |  |
| A1R5X6 | D-ribose-binding protein | 1.808 | 0.003 | 7.307 | 0.005 |
| A1RDL9 | Putative major facilitator superfamily (MFS) transporter | 2.285 | 0.029 | 2.490 | 0.031 |
| A1R1Q2 | D-ribose-binding protein | 2.253 | 0.024 | 1.962 | 0.013 |
| A1R321 | Phosphomannomutase | 1.929 | 0.013 | 1.526 | 0.019 |
| A1R6X7 | Glycerol uptake facilitator protein | 1.884 | 0.016 | 1.541 | 0.040 |
| A1RAD7 | Hydroxypyruvate isomerase | 1.496 | 0.038 | 2.670 | 0.018 |
| A1R437 | Putative sugar ABC transporter permease protein | 4.909 | 0.026 | 2.173 | 0.020 |
| A1R6U1 | Phosphocarrier protein HPr | 3.002 | 0.016 | 2.510 | 0.012 |
|  | **Cell cycle control, cell division, chromosome partitioning** |  |  |  |  |
| A1R9E9 | Putative cell surface polysaccharide biosynthesis | 1.516 | 0.004 | 1.751 | 0.039 |
|  | **Cell motility** |  |  |  |  |
| A1RCA7 | Putative ParA-family protein | 2.326 | 0.042 | 1.920 | 0.017 |
|  | **Cell wall/membrane/envelope biogenesis** |  |  |  |  |
| A1R5T0 | Prolipoprotein diacylglyceryl transferase | 1.877 | 0.020 | 3.997 | 0.031 |
| A1RBS3 | Large conductance mechanosensitive channel protein | 1.793 | 0.050 | 1.465 | 0.040 |
| A1R7L6 | Putative ABC transporter, ATP-binding protein | 2.351 | 0.047 | 1.512 | 0.036 |
|  | **Coenzyme transport and metabolism** |  | 0.028 |  |  |
| A1R5J6 | Octanoyltransferase | 1.655 |  |  |  |
| A1R5E5 | Putative polyprenyl synthetase | 2.108 | 0.017 | 1.705 | 0.046 |
| A1R8A5 | Uroporphyrinogen decarboxylase | 1.604 | 0.010 | 1.987 | 0.036 |
| A1R7A9 | Putative OsmC-like protein | 1.329 | 0.012 | 2.282 | 0.022 |
|  | **Function unknown** |  |  |  |  |
| A1R973 | Uncharacterized protein | 1.541 | 0.036 | 1.981 | 0.021 |
| A1RAR5 | Putative DGPF domain protein | 2.363 | 0.044 | 1.912 | 0.024 |
| A1R1S2 | Putative membrane protein (DUF445) | 4.560 | 0.011 | 2.717 | 0.050 |
| A1R1S7 | Uncharacterized protein | 1.921 | 0.010 | 2.355 | 0.019 |
|  | **General function prediction only** |  |  |  |  |
| A1R8K1 | Uncharacterized protein | 3.595 | 0.031 | 2.204 | 0.024 |
| A1R4N1 | Oxidoreductase, short chain dehydrogenase/reductase family | 1.500 | 0.004 | 2.658 | 0.005 |
| A1R6K3 | Oxidoreductase, short chain dehydrogenase/reductase family | 1.441 | 0.012 | 1.405 | 0.019 |
| A1R854 | Putative methanol dehydrogenase regulatory protein (MoxR2) | 5.335 | 0.013 | 4.270 | 0.013 |
|  | **Inorganic ion transport and metabolism** |  |  |  |  |
| A1RC00 | Copper-translocating P-type ATPase | 3.427 | 0.010 | 3.367 | 0.033 |
| A1R4C0 | Carbonic anhydrase | 1.414 | 0.036 | 1.414 | 0.024 |
| A1R375 | Phosphate-specific transport system accessory protein PhoU | 2.957 | 0.025 | 3.014 | 0.021 |
| A1R7B2 | Protozoan/cyanobacterial globin-like family protein | 3.873 | 0.020 | 3.807 | 0.005 |
|  | **Lipid transport and metabolism** |  |  |  |  |
| A1R7F3 | Acyl carrier protein | 1.518 | 0.005 | 1.991 | 0.012 |
| A1R5P3 | Putative acetyl-coa acetyltransferase (Thiolase) | 1.662 | 0.016 | 1.627 | 0.026 |
| A1R246 | Enoyl-CoA hydratase/isomerase family protein | 1.914 | 0.025 | 3.077 | 0.005 |
| A1R4N0;A1R7E7 | Propionyl-CoA carboxylase complex B subunit | 2.261 | 0.012 | 1.968 | 0.038 |
|  | **Nucleotide transport and metabolism** |  |  |  |  |
| A1R108 | Uracil-xanthine permease | 2.715 | 0.029 | 4.566 | 0.012 |
|  | **Posttranslational modification, protein turnover, chaperones** |  |  |  |  |
| A1R6S1 | Putative integral membrane protein | 3.229 | 0.050 | 2.068 | 0.024 |
| A1RCV2 | Putative Thioredoxin domain protein (DSBA) | 2.209 | 0.004 | 2.221 | 0.020 |
| A1R8M2 | 10 kDa chaperonin | 2.645 | 0.017 | 2.100 | 0.020 |
| A1RCA2 | Thioredoxin reductase | 2.171 | 0.006 | 1.542 | 0.019 |
| A1RB15 | Putative NHL repeat protein | 1.640 | 0.004 | 2.177 | 0.007 |
| A1RAE3 | Bacterioferritin comigratory protein (Thioredoxin reductase) | 1.482 | 0.012 | 2.027 | 0.020 |
| A1R5Q3 | SufE protein | 2.549 | 0.046 | 1.648 | 0.035 |
| A1R1W2 | Proteasome subunit beta | 5.470 | 0.008 | 5.963 | 0.006 |
| A1R8X6 | Putative aminomethyltransferase (Glycine cleavage system Tprotein) | 2.095 | 0.004 | 2.157 | 0.037 |
|  | **Signal transduction mechanisms** |  |  |  |  |
| A1R884 | Uncharacterized protein | 1.585 | 0.048 | 3.683 | 0.005 |
| A1R1M9 | Putative EAL domain protein | 3.072 | 0.050 | 1.562 | 0.024 |
|  | **Transcription** |  |  |  |  |
| A1R6Y9 | DNA-directed RNA polymerase subunit omega | 1.625 | 0.002 | 1.449 | 0.049 |
| A1R9V6 | Putative transcriptional regulator, lacI family | 4.669 | 0.043 | 4.570 | 0.018 |
| A1R2G6 | Transcription regulator, GntR-family | 2.703 | 0.012 | 3.047 | 0.012 |
| A1R8W3 | Transcription termination/antitermination protein NusG | 1.479 | 0.012 | 1.803 | 0.012 |
| A1R316 | Transcriptional regulator, GntR-family | 3.186 | 0.016 | 3.021 | 0.017 |
| A1RA38 | Transcriptional regulator for glyoxylate bypass, IclR family | 1.454 | 0.010 | 1.338 | 0.026 |
| A1R620 | Putative transcriptional regulator, MarR family | 1.739 | 0.019 | 1.454 | 0.048 |
| A1RCZ4 | Uncharacterized protein | 1.385 | 0.034 | 1.533 | 0.030 |
| A1R5L5 | Putative transcriptional regulator, BlaI/MecI/CopY family | 1.662 | 0.045 | 2.048 | 0.020 |
| A1R7Z7 | Putative RNA polymerase sigma (70) factor | 2.813 | 0.034 | 3.354 | 0.017 |
|  | **Translation, ribosomal structure and biogenesis** |  |  |  |  |
| A1R6V9 | Endoribonuclease YbeY | 1.603 | 0.020 | 1.457 | 0.040 |
| A1R568 | 50S ribosomal protein L20 | 2.315 | 0.037 | 1.390 | 0.034 |
| A1R7W5 | Peptide chain release factor 1 | 1.709 | 0.006 | 2.184 | 0.023 |
| A1R7C4 | Peptide deformylase | 2.783 | 0.048 | 1.745 | 0.019 |
| A1R2N3 | Acetyltransferase, GNAT family protein | 2.197 | 0.003 | 3.283 | 0.012 |
| A1R8N1 | Ribosomal-protein-alanine acetyltransferase | 2.421 | 0.012 | 2.356 | 0.018 |
|  | **Unknown** |  |  |  |  |
| A1R9K1 | Putative S-layer domain protein | 1.389 | 0.042 | 1.456 | 0.031 |
| A1RBA4 | FG-GAP repeat domain protein | 2.129 | 0.008 | 1.548 | 0.027 |
| A1R4M4 | Putative integral membrane protein, MMPL family | 2.991 | 0.004 | 2.371 | 0.015 |
| A1R5Q7 | Uncharacterized protein | 2.340 | 0.012 | 1.785 | 0.029 |
| A1R7Z6 | Putative anti-sigma factor | 1.591 | 0.044 | 1.912 | 0.024 |
| A1R5L9 | Uncharacterized protein | 3.818 | 0.034 | 3.739 | 0.031 |
| A1RAG1 | Uncharacterized protein | 2.159 | 0.013 | 1.833 | 0.025 |
| A1R5M8 | Uncharacterized protein | 2.557 | 0.017 | 2.818 | 0.017 |
| A1R4M1 | Putative major facilitator superfamily (MFS) transporter | 2.917 | 0.005 | 2.581 | 0.012 |
| A1R7T7 | Uncharacterized protein | 1.755 | 0.024 | 1.811 | 0.023 |
| A1R9R2 | Uncharacterized protein | 3.184 | 0.013 | 2.750 | 0.024 |
| A1R9T5 | Uncharacterized protein | 3.456 | 0.003 | 2.743 | 0.012 |
| A1R2Z1 | Putative transcriptional regulatory protein | 1.877 | 0.029 | 1.793 | 0.030 |
| A1RCR1 | Uncharacterized protein | 1.935 | 0.016 | 1.706 | 0.025 |
| A1RCM0 | Uncharacterized protein | 1.373 | 0.031 | 2.464 | 0.007 |
| A1R9W0 | Putative transcriptional regulator, ArsR family | 1.872 | 0.030 | 2.125 | 0.021 |
| A1R4X5 | Uncharacterized protein | 1.569 | 0.045 | 1.560 | 0.038 |
| A1RC02 | Uncharacterized protein | 2.500 | 0.007 | 1.492 | 0.039 |
| A1R6A6 | Uncharacterized protein | 1.971 | 0.012 | 1.415 | 0.034 |
| A1R6R4 | Sec-independent protein translocase protein TatA | 3.351 | 0.002 | 2.138 | 0.017 |
| A1RA26 | Acetyltransferase, GNAT family protein | 4.213 | 0.003 | 2.625 | 0.019 |
| A1RAE4 | Two-component system response regulator | 1.918 | 0.007 | 1.454 | 0.023 |
| A1RD78 | BNR/Asp-box repeat domain protein | 1.941 | 0.016 | 1.324 | 0.049 |
| A1RAE7 | Uncharacterized protein | 1.582 | 0.035 | 1.565 | 0.032 |
| A1RCQ2 | BNR/Asp-box repeat domain protein | 2.006 | 0.042 | 2.048 | 0.033 |
| A1RAV0 | Uncharacterized protein | 2.030 | 0.015 | 3.100 | 0.006 |
| A1R0V2 | Putative FHA domain protein | 1.538 | 0.050 | 1.456 | 0.049 |
| A1R2W0 | Uncharacterized protein | 1.508 | 0.042 | 1.401 | 0.042 |
| A1R826 | Putative integral membrane protein | 2.057 | 0.037 | 2.279 | 0.026 |
| A1RD31 | Uncharacterized protein | 1.486 | 0.048 | 1.783 | 0.026 |
| **Downregulated proteins** | |  |  |  |  |
|  | **Amino acid transport and metabolism** |  |  |  |  |
| A1R2I8 | Putative glycine betaine/L-proline transport ATP binding subunit | -3.909 | 0.007 | -3.276 | 0.019 |
|  | **Defense mechanisms** |  |  |  |  |
| A1R327 | Putative 5'-nucleotidase family protein | -1.687 | 0.036 | -1.812 | 0.028 |
| A1R510 | Streptomycin 6-kinase | -2.645 | 0.007 | -1.588 | 0.039 |
|  | **General function prediction only** |  |  |  |  |
| A1R4Z0 | Oxidoreductase family, NAD-binding Rossmann fold domain protein | -2.120 | 0.014 | -2.179 | 0.018 |
|  | **Posttranslational modification, protein turnover, chaperones** |  |  |  |  |
| A1R5W7 | Chaperone protein ClpB | -4.771 | 0.004 | -3.431 | 0.018 |
|  | **Translation, ribosomal structure and biogenesis** |  |  |  |  |
| A1R5E9 | Transcriptional regulator MraZ | -2.065 | 0.002 | -1.446 | 0.012 |
|  | **Unknown** |  |  |  |  |
| A1R780 | Putative cupin domain protein | -3.251 | 0.004 | -2.641 | 0.012 |
| A1R864 | Putative ferredoxin reductase | -4.974 | 0.002 | -3.128 | 0.017 |
| A1R3T7 | CBS domains protein | -5.794 | 0.001 | -2.003 | 0.045 |

**References**

1. Nelder JA, Mead R. A Simplex Method for Function Minimization. *Comput J* 1965; **7**: 308–313.

2. Brun R, Reichert P, Künsch HR. Practical identifiability analysis of large environmental simulation models. *Water Resour Res* 2001; **37**: 1015–1030.

3. Oliphant TE. Python for Scientific Computing. *Comput Sci Eng* 2007; **9**: 10–20.

4. Strong LC, Rosendahl C, Johnson G, Sadowsky MJ, Wackett LP. Arthrobacter aurescens TC1 metabolizes diverse s-triazine ring compounds. *Appl Environ Microbiol* 2002; **68**: 5973–5980.

5. Speight JG, others. Lange’s handbook of chemistry. 2005. McGraw-Hill New York.

6. Schwarzenbach RP, Gschwend PM, Imboden DM. Environmental Organic Chemistry 2003. Wiley: Hoboken, NJ.

7. Ehrl BN, Kundu K, Gharasoo M, Marozava S, Elsner M. Rate-Limiting Mass Transfer in Micropollutant Degradation Revealed by Isotope Fractionation in Chemostat. *Environ Sci Technol* 2019; **53**: 1197–1205.

8. Bradford MM. A rapid and sensitive method for the quantitation of microgram quantities of protein utilizing the principle of protein-dye binding. *Anal Biochem* 1976; **72**: 248–254.

9. Shapir N, Mongodin EF, Sadowsky MJ, Daugherty SC, Nelson KE, Wackett LP. Evolution of catabolic pathways: genomic insights into microbial s-triazine metabolism. *J Bacteriol* 2007; **189**: 674–682.

10. Erickson LE, Lee KH, Sumner DD. Degradation of atrazine and related s-triazines. *Crit Rev Environ Sci Technol* 1989; **19**: 1–14.

11. Zeng Y, Sweeney C, Stephens S, Kotharu P, Turnbull M. Atrazine Pathway Map. http://eawag-bbd.ethz.ch/atr/atr_map.html. .

12. Mongodin EF, Shapir N, Daugherty SC, DeBoy RT, Emerson JB, Shvartzbeyn A, et al. Secrets of soil survival revealed by the genome sequence of Arthrobacter aurescens TC1. *PLoS Genet* 2006; **2**: e214.

13. Love MI, Huber W, Anders S. Moderated estimation of fold change and dispersion for RNA-seq data with DESeq2. *Genome Biol* 2014; **15**: 550.
